# CO_2_ Hydrogenation on Ru Single-Atom Catalyst Encapsulated in Silicalite: a DFT and Microkinetic Modeling Study

**DOI:** 10.1021/acs.jpcc.4c05941

**Published:** 2024-09-23

**Authors:** Manuel A. Cánovas, Alejandro Gracia, Ramón Sayós, Pablo Gamallo

**Affiliations:** Departament de Ciència de Materials i Química Física & Institut de Química Teòrica i Computacional (IQTCUB), Universitat de Barcelona, C. Martí i Franquès, 1, 08028 Barcelona, Spain

## Abstract

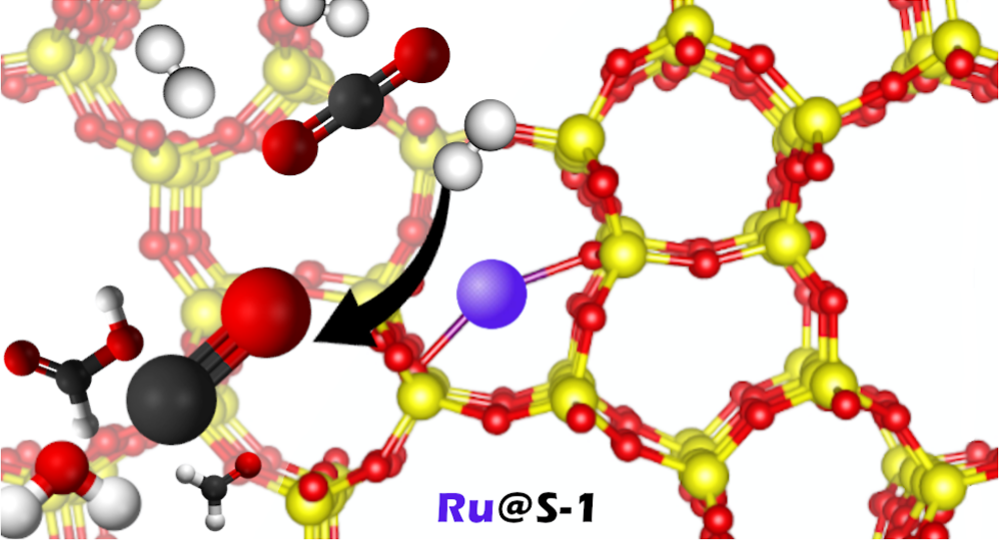

The critical levels of CO_2_ emissions reached in the past decade have encouraged researchers into finding techniques to reduce the amount of anthropogenic CO_2_ expelled to the atmosphere. One possibility is to capture the produced CO_2_ from the source of emission or even from air (i.e., direct air capture) by porous materials (e.g., zeolites and MOFs). Among the different usages of captured CO_2_, its conversion into light fuels such as methane, methanol, and formic acid is essential for ensuring the long-awaited circular economy. In the last years, single-atom catalysts encapsulated in zeolites have been considered to this purpose since they exhibit a high selectivity and activity with the minimum expression of catalytic species. In this study, a detailed mechanism composed by 47 elementary reactions, 42 of them in both forward and reverse directions and 5 of them that correspond to the desorption of gas products just forwardly studied), has been proposed for catalytic CO_2_ hydrogenation over Ru SAC encapsulated in silicate (Ru_1_@S-1). Periodic density functional theory (DFT) calculations along with microkinetic modeling simulations at different temperatures and pressures were performed to evaluate the evolution of species over time. The analysis of the results shows that carbon monoxide is the main gas produced, followed by formic acid and formaldehyde. The rate analysis shows that CO_(g)_ is formed mainly through direct dissociation of CO_2_ (i.e., redox mechanism), whereas COOH formation is assisted by OH. Moreover, the Campbell’s degree of rate control analysis suggests that the determining steps for the formation of CO_(g)_ and CH_2_O_(g)_ gas species are their own desorption processes. The results obtained are in line with recent experimental and theoretical results showing that Ru_1_ SACs are highly selective to CO_(g)_, whereas few atom clusters as Ru_4_ increase selectivity toward methane formation.

## 1 Introduction

Over the last century, there has been an exponential increase in CO_2_ emissions into the atmosphere due to the high dependence of fossil fuels in different sectors, such as transport and industry. Nowadays, it is well-known that combustion of fossil fuels plays a key role in the climate change and rise of global temperature. For this reason, in the past decade, CO_2_ capture and conversion into light fuels such as methane, methanol, formaldehyde, and formic acid, among others, has become a relevant topic of investigation. Many transition metal (TM)-based catalysts have been explored for their use in the CO_2_ hydrogenation process (e.g., primarily non-noble metals like Ni, Cu, and Fe^1−3^ as well as noble metals such as Au, Ru, Rh, Pd, and Pt^4−7^). However, better catalytic systems need to be found as some of the TMs exposed before are exotic and expensive.

In the past few years, single-atom catalysts (SACs) have attracted the attention of research groups since they exhibit the highest activity per active site along with a good selectivity at a lower price.^8,9^ However, SACs’ major drawbacks are the sintering and aggregation into nanoparticles that makes them lose their positive attributes. For this reason, finding a suitable support that stabilizes the SAC is as important as choosing the SAC itself. Within this frame of reference, zeolites are vastly used as supports for small particles (including SAC and nanoparticles) as their porous structure provides a good confinement against sintering. Moreover, the selectivity of zeolites directly correlates with the size of the molecules that can fit into the pore.^10−14^ Thus, zeolites have been used in several industrial applications such as postcombustion CO_2_ capture and fuel processing.^15^

Ru-based catalysts have been used for enhancing a huge variety of reactions. For example, the Ru_1_/mpg-C_3_N_4_ catalyst shows phenomenal hydrogenation and hydrodeoxygenation selectivity and performance for vanillin hydrogenation to vanillyl alcohol.^16^ Hung et al. showed that Ru exhibits a high CO affinity over competing H, suggesting that they are a good candidate for CO_2_ methanation.^17^ Wu et al. studied the CO_2_ hydrogenation to methanol on Ru/In_2_O_3_ showing that the high activity in the hydrogenation is caused by the enhanced CO_2_ adsorption and hydrogen spillover effect.^18^ Sun et al. studied the water–gas shift (WGS) reaction on RuFeO_*x*_, concluding that single Ru atoms do not accomplish methanation when Ru_1_ is on surface due to a low CO adsorption along with an easy desorption of H_2_.^19^ Besides, ultrafine Ru clusters were encapsulated into ZSM-5 zeolites,^20^ showing an increase in activity and stability for the hydrodeoxygenation of phenol to cyclohexane, a crucial reaction in biomass valorization. Moreover, Kwak et al. studied the catalytic performance of small Ru clusters in Ru/Al_2_O_3_ catalysts for the CO_2_ reduction and conversion into CH_4_,^21^ and similarly, Wang et al. studied the CO_2_ methanation on Ru/CeO_2_ and Ru/SiO_2_.^22^ Finally, small clusters of Ru_*n*_/Ru(0001) (*n* = 1–4 atoms) were used in CO_2_ methanation, showing promising results with high selectivity to CO and CH_4_ for *n* = 1 and *n* = 4, respectively.^23^ CO_2_ hydrogenation on Ru-based catalysts generally favors high temperatures and moderate pressures. For example, the studies cited indicate that CO_2_ reduction to methane (CH_4_) on Ru catalysts occurs efficiently at temperatures from 400 to 650 K and pressures ranging from 1 to 10 atm (0.1–1 MPa).

In this study, silicalite (S-1) is used for encapsulating a single Ru atom (i.e., Ru_1_@S-1). S-1 is easy to synthesize,^24^ and recently, Ru SAC has been successfully encapsulated experimentally as Ru_1_@S-1,^25^ exhibiting a higher selectivity and activity in ammonia synthesis than pristine zeolite. Moreover, Ru nanoparticles have been also encapsulated in S-1 obtaining high selectivity toward methane, especially when silicalite is treated with NaOH.^26^

This study focuses on CO_2_ hydrogenation on the Ru_1_@S-1 catalyst for assessing the viability of using single Ru atoms in the CO_2_ conversion. According to refs (27,28), published some years ago by some of us, Ru_1_@S-1 could be a promising catalyst for CO_2_ hydrogenation due to the low energy barrier associated with the CO_2_ activation along with a low activity expected over the sintering of Ru atoms.

## 2 Methodology and Computational Details

### 2.1 DFT Calculations

**Figure 1:**
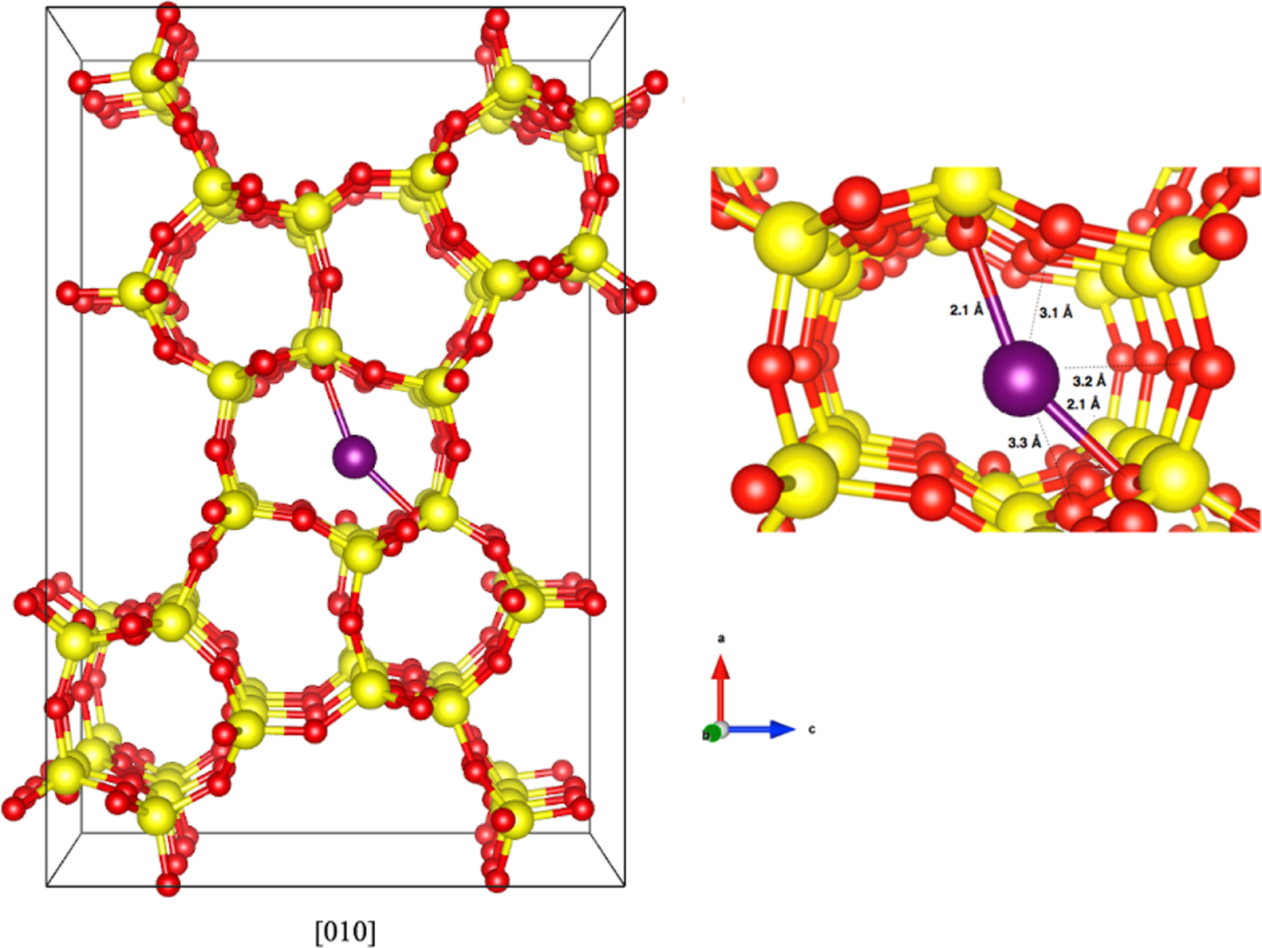
Most stable adsorption site for Ru atom in S-1 according to ref (27). The Ru atom is encapsulated inside a six-membered ring and binds with two pore wall O atoms at 2.1 Å. Si, O, and Ru atoms are represented with yellow, red, and purple spheres, respectively. The unit cell is shown with continuous lines.

MFI silicalite-1 (S-1) is a porous zeolite with the molecular formula Si_96_O_192_. The crystallographic position of the atoms in S-1 was obtained from the Zeolite Structure Database,^29^ while the most suitable location of the 29 different TM atoms in S-1 (TM_1_@S-1) was previously studied by means of DFT calculations.^27^ Moreover, Alonso et al.^28^ studied CO_2_ conversion into CO directly (redox pathway) or through H-assisted (formate and carboxyl) pathways for the TMs that presented high aggregation resistance as well as good catalytic performance over CO_2_ activation (e.g., Ni, Ru, Rh, Pd, and Pt). The study concluded that among these TMs, Ru and Rh are the best candidates for CO_2_ hydrogenation, and specifically, Ru_1_@S-1 shows the smallest energy barrier and highest (more negative) adsorption energy for the redox mechanism. Figure 1 shows the Ru_1_@S-1 unit cell with the Ru atom located at the most stable site, according to DFT calculations. The site corresponds to the Ru atom located inside the central pore and closely coordinated with two oxygen atoms at Ru–O distances of 2.1 Å and weakly coordinated to other two oxygen atoms at Ru–O distances of 3.1 Å, distances that are in agreement with the experimental results.^30^ Moreover, the adsorption energy of Ru on this site is −0.65 eV, a value that preserves sintering at the moderate operating temperatures considered in this study.^27,28^

Spin-polarized periodic DFT calculations have been performed by means of the Vienna Ab initio Simulation Package (VASP)^31^ and using the Perdew–Burke–Ernzerhof^32^ exchange–correlation functional, alongside the Grimme D3 dispersion correction (PBE-D3).^33^ The electron density valence was expanded on a plane-wave basis with a limitation of 600 eV in the kinetic energy. Owing to the large size of the optimized DFT values of the unit cell [20.30 × 19.86 × 13.37 Å^3^ with 289 atoms (Ru@Si_96_O_192_)],^27^ the Brillouin zone was sampled only in the Γ-point. The effect of the core electrons on the valence electron density was reckoned through the projected augmented wave (PAW) method,^34,35^ which also includes scalar relativistic effects. The energy and force tolerance were set at 10^–5^ eV and 0.01 eV/Å, respectively.

Several positions and orientations over the Ru atom have been studied for all of the species considered. The selected adsorption site was the most stable location after ensuring that the stationary point corresponded to a minimum by means of a frequency analysis. Adsorption energies (Δ*E*_ads,*i*_) for the *i*-species are calculated as
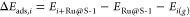
1where *E*_*i*+Ru@S-1_ is the energy of the *i*-species adsorbed in Ru_1_@S-1, *E*_Ru@S-1_ corresponds to the energy of the pristine Ru_1_@S-1, and *E*_*i*(*g*)_ is the energy for the *i*-species in the gas phase. According to this, a negative adsorption energy is favorable as the adsorbate is more stable in the zeolite than in the gas phase. All reported energy values have been zero-point energy (ZPE) corrected. Moreover, energy barriers (Δ*E*^≠^) and reaction energies (Δ*E*_r_) have been obtained as
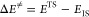
2
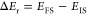
3where *E*_IS_, *E*_FS_, and *E*^TS^ correspond to the ZPE corrected energy values for the initial state (IS), the final state (FS), and the transition state (TS), respectively, for any elementary reaction considered. TSs were obtained through the climbing-image nudged elastic band (CI-NEB) method.^36^ It consists of an iterative technique in which the minimum energy pathway (MEP) between IS and FS is found using intermediate images generated by means of the image-dependent pair potential (IDPP) interpolation procedure,^37^ as implemented in the atomic simulation environment (ASE) package.^38^ In the case of more than one imaginary frequency, the Dimmer technique was also applied.^39,40^ Thus, all TSs were confirmed by vibrational analysis, ensuring one single imaginary frequency.

However, this iterative procedure proved to be extremely slow and highly computationally demanding as all the intermediate images are evaluated and optimized in every step to fulfill both energy and force tolerances. In addition, the force optimization is very computationally expensive, and sometimes, the force tolerance for the TS is unreachable using CI-NEB for big systems like Ru_1_@S-1. Fortunately, in the past few years, a new computational technique based on surrogate machine learning^41,42^ has received attention as it reduces drastically the high computational cost of the CI-NEB method. Explaining this method is beyond the scope of this project. Nevertheless, it is worth noting that the intermediate images are computed as single points rather than as optimizations. This procedure decreases the computational cost and helps us find complex TSs like those related to HCOO formation from CO_2_ assisted by OH and H_2_O.

Finally, the Gibbs free energy for the entire set of reactions was obtained as
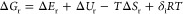
4where Δ*U*_r_ and Δ*S*_r_ are the variations in internal energy and entropy, respectively, associated with the reaction, and δ_i_ is a factor equal to −1 for an adsorption, +1 for a desorption, and 0 for the activated conversion of the adsorbed reactants (i.e., Langmuir–Hinshelwood reaction: X* + Y*→ Z_(g)_ + 2*, where * indicates adsorption sites). This term comes from the Δ(*PV*) assuming ideal gas for gas-phase species as well as this variation being negligible for adsorbed reactants species. Notice that the zero-reference point was set at the first vibrational level. For this reason, Δ*U*_r_ was calculated without ZPE, but it is included in the Δ*E*_r_ term, defined in eq 3. Equations for Δ*U*_r_ and Δ*S*_r_ can be found in ref (43). Note that we established a cutoff in the frequencies of 6.9 meV, which means that if any frequency is below the cutoff, it is raised to 6.9. This has been done in similar studies^2,44^ to prevent small frequencies to give extremely large contributions to vibrational partition functions, hence to the Δ*G*_r_.

### 2.2 MkM Calculations

**Figure 2:**
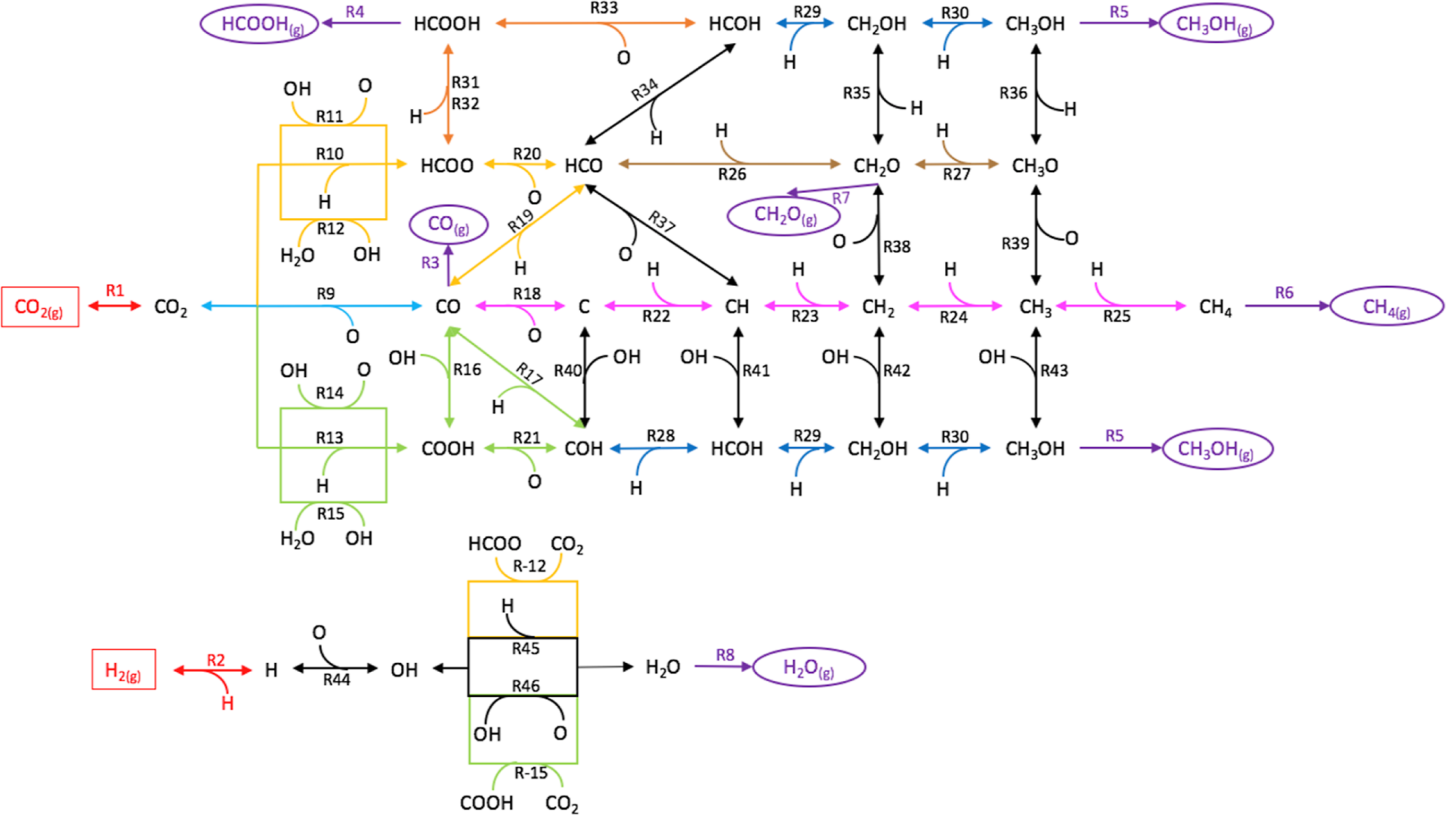
Reaction pathways proposed for CO_2_ hydrogenation (top) and H_2_ oxidation (bottom). Red and purple lines denote reactants adsorption/desorption and products desorption, respectively. The redox mechanism (blue), the assisted formate dissociation mechanism (yellow), and the assisted carboxyl dissociation mechanism (green) constituted the reverse WGS reaction. The C, HCO, and COH hydrogenation pathways are represented by pink, brown, and dark blue lines and constituted the CH_4(g)_, CH_2_O_(g)_, and CH_3_OH_(g)_ formation reactions, respectively. Orange lines denote formic acid formation and black lines indicate connectivity between pathways. Reversible reactions are represented by double arrows. The minus sign (e.g., R-15) designates the backward reaction.

Microkinetic modeling (MkM) provides the time evolution dependence of gas species production and the effect of temperature and pressure on it. To do so, it is necessary to propose possible reaction pathways for the CO_2_ hydrogenation and H_2_ oxidation, which are presented in Figure 2. The total reaction network contains 47 reversible elementary reactions, which account for several gas products (i.e., CO_(g)_, HCOOH_(g)_, CH_3_OH_(g)_, CH_4(g)_, CH_2_O_(g)_, and H_2_O_(g)_). In this study, only Langmuir–Hinshelwood reactions have been considered, so if there is more than one species in the IS or FS, a co-adsorption on the Ru atom is required. The re-adsorption of these gas products has not been considered, assuming a plug flow reactor model.

The parameters for the microkinetic model are based on the DFT data. In the present study, the adsorption energies of gas species are nonactivated, and thus, the rate constant associated with these processes (*k*_ads,*i*_) have been obtained according to the Hertz-Knudsen expression and similarly, for the desorption rate constants (*k*_des,*i*_). Finally, for the activated conversion of the adsorbed reactants, the conventional transition state theory (TST) rate constant equation (*k*_TST_) was used. All of these expressions for the rate constants can be found in Section S1 in Supporting Information.

Once all rate constants have been obtained, it is necessary to solve the following system of differential equations
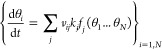
5where θ_*i*_ is the coverage of the *i*-species at a time *t*, *v*_*ij*_ is the stoichiometric coefficient of the *i*-species in elementary reaction *j*, *k*_*j*_ is the rate constant for reaction *j*, and *f*_*j*_(θ_1_...θ_*N*_) is a function of all the coverages contributing to reaction *j*. Notice that if the *i*-species is not involved in the *j*-reaction, it does not contribute to θ_*i*_. More details about the procedure can be found in ref (45). MkM simulations were carried out using MKMCXX^46^ code that has been already used in similar zeolitic systems.^23,47−49^ Furthermore, Campbell’s degree of rate control (DRC,^50^*X*_RC,*i*_) has been used to measure the contribution of any elementary step over the entire reaction rate and to determine the rate-determining step (RDS) reaction that usually corresponds to the largest *X*_RC,*i*_ value.

Finally, the gas production rate or turnover frequency (TOF) (*r*_*i*_) for the *i*-th species at several temperatures were obtained, and then, the apparent activation energy (*E*_a,*i*_) associated with the *i*-th species production was obtained through the following Arrhenius equation
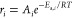
6where *A*_*i*_ is a pre-exponential factor.

In the MkM simulations, a pristine Ru_1_@S-1 structure interacts with an ideal H_2_/CO_2_ gas mixture in a 4:1 ratio at different pressures (i.e., 1, 5, and 10 bar) and temperatures in the range of 300–800 K, as are the normal experimental and computational conditions for the RWGS reactions.^1,2,4−7,23,51,52^ The initial gas mixture ratio was chosen to allow the system to form all possible gas products studied (e.g., CH_4(g)_) and not favoring any of them. In addition, not increasing the temperature over 800 K ensures a desirable stability of the SAC without the aggregation and cluster formation.^25^ Simulations were carried out until reaching steady state.

## 3 Results and Discussion

### 3.1 DFT Results

#### 3.1.1 Gas Adsorption and Desorption Processes

According to Figure 2, there are a total of 8 gas-phase species and 22 adsorbed species in the total reaction network. First, the adsorption energy for all of the reactant species in Ru_1_@S-1 was obtained, also evaluating the optimal geometries in both gas and adsorbed species (Table S1 of Supporting Information). In general, adsorbed species have larger bonds than in the gas-phase. The configuration for adsorbed species is shown in Figure S1 in Supporting Information. Remarkably, H_2_ spontaneously dissociates when it adsorbs, deriving into a co-adsorption of 2H atoms over the Ru active site. Adsorbed CO_2_ loses its linear form, becoming bent to an angle of 141°. In the case of CH_2_O, the C–O bond enlarges when it adsorbs to the Ru atom. Regarding methane, the C–H distances are retained like those in gas-phase, except one of them that enlarges. For HCOOH and CH_3_OH species, the interaction with the Ru atom is through the oxygen atom of CO and the OH group, respectively (Figure S1 in Supporting Information).

Table 1 shows the ZPE-corrected adsorption and desorption energies for all of the species at the most stable configuration. In the case that two species are implied in the reaction, the DFT energies have been obtained as co-adsorbed species. CO_2_ and H_2_ adsorptions correspond to strong chemisorption processes. The CO desorption process (R3) exhibits a high desorption energy of 3.30 eV. Such a strong adsorption does not favor CO_(g)_ formation and produces a high CO coverage. However, from a catalytic point of view, it enables adsorbed CO species to interact with other species through more favorable reactive processes. To an extent, this can also be applied to the CH_2_O desorption process (R7) that with an energy barrier of 2.44 eV, which corresponds to the endothermicity, behaves similarly to CO desorption. Desorbing the other possible gas products is expected to be more feasible from an energetic point of view since the energy barriers associated are lower than 1 eV. However, it is necessary to consider not only the direct adsorption and desorption processes but also the different routes to them.

**Table 1:** Reaction Energies (Δ*E*_*r*_) and Energy Barriers (Δ*E*^≠^) for All Elementary Reactions Shown in Figure 2, with Zero-Point Energy Already Included^a^

reactions	Δ*E*_r_	Δ*E*^≠^	reactions	Δ*E*_r_	Δ*E*^≠^
R1: CO_2(g)_ + * ⇌ CO_2_	–1.63	0.00	R25: CH_3_ + H ⇌ CH_4_ + *	0.63	0.63
R2: H_2(g)_ + 2* ⇌ 2H	–1.52	0.00	R26: HCO + H ⇌ CH_2_O + *	0.25	0.85
R3: CO ⇌ CO_(g)_ + *	3.30	3.30	R27: CH_2_O + H ⇌ CH_3_O + *	0.67	1.28
R4: HCOOH → HCOOH_(g)_ + *	1.07	1.07	R28: COH + H ⇌ HCOH + *	0.56	1.12
R5: CH_3_OH → CH_3_OH_(g)_ + *	1.09	1.09	R29: HCOH + H ⇌ CH_2_OH + *	1.00	1.00
R6: CH_4_ → CH_4(g)_ + *	0.63	0.63	R30: CH_2_OH + H ⇌ CH_3_OH + *	0.58	1.15
R7: CH_2_O → CH_2_O_(g)_ + *	2.44	2.44	R31: b-HCOO + H ⇌ HCOOH + 2*	1.58	1.58
R8: H_2_O → H_2_O_(g)_ + *	0.77	0.77	*R*32: m-HCOO + H ⇌ HCOOH + *	1.10	1.39
R9: CO_2_ + * ⇌ CO + O	–0.61	0.48	R33: HCOH + O ⇌ HCOOH + *	0.69	1.35
R10: CO_2_ + H ⇌ b-HCOO	0.65	1.43	R34: HCO + H ⇌ HCOH + *	0.83	1.60
R11: CO_2_ + OH + * ⇌ b-HCOO + O	0.13	2.15	R35: CH_2_O + H ⇌ CH_2_OH + *	1.26	1.55
R12: CO_2_ + H_2_O + * ⇌ b-HCOO + OH	–0.03	1.79	R36: CH_3_O + H ⇌ CH_3_OH + *	1.08	1.41
R13: CO_2_ + H ⇌ b-COOH	0.91	1.78	R37: HCO + * ⇌ CH + O	0.58	2.29
R14: CO_2_ + OH + * ⇌ b-COOH + O	0.47	0.84	R38: CH_2_O + * ⇌ CH_2_ + O	0.32	1.80
R15: CO_2_ + H_2_O + * ⇌ b-COOH + OH	0.12	0.45	R39: CH_3_O + * ⇌ CH_3_ + O	–0.69	1.40
R16: b-COOH ⇌ CO + OH	–1.88	0.58	R40: COH + * ⇌ C + OH	–0.19	1.57
R17: CO + H ⇌ COH + *	2.18	3.08	R41: HCOH + * ⇌ CH + OH	0.08	1.20
R18: CO + * ⇌ C + O	2.57	4.01	R42: CH_2_OH + * ⇌ CH_2_ + OH	–1.03	0.80
R19: CO + H ⇌ HCO + *	1.93	1.99	R43: CH_3_OH + * ⇌ CH_3_ + OH	–1.67	1.12
R20: b-HCOO ⇌ HCO + O	0.57	1.50	R44: O + H ⇌ OH + *	0.24	1.09
R21: b-COOH ⇌ COH + O	0.49	1.78	R45: OH + H ⇌ H_2_O + *	1.11	1.38
R22: C + H ⇌ CH + *	0.48	1.37	R46: OH + OH ⇌ H_2_O + O	0.83	1.03
R23: CH + H ⇌ CH_2_ + *	0.36	0.96	R47: b-HCOO ⇌ *m*-HCOO + *	0.37	0.56
R24: CH_2_ + H ⇌ CH_3_ + *	0.03	0.77			

a Values are in eV. An asterisk represents a free site considering that two species can adsorb on the Ru atom. R47 refers to the change of denticity for HCOO from bidentate to monodentate.

#### 3.1.2 Products Formation

Figure 2 shows the three different routes available to obtain CO, namely, the direct redox dissociation (R9, Δ*E*^≠^ = 0.48 eV) along with the two possible assisted CO formations, one through HCOO (R10–R12, Δ*E*^≠^ = 1.43, 2.15, and 1.79 eV, respectively) and another through COOH (R13–R15, Δ*E*^≠^ = 1.78, 0.84, and 0.45 eV, respectively). Each assisted CO formation was studied by H, OH, and H_2_O separately. Initially, one can expect that the redox reaction (R9) dominates because of its exoergicity (Δ*E*_r_ = −0.61 eV) and the small energy barrier, as anticipated by Alonso and co-workers.^28^ The HCOO intermediate was found in two species, as monodentate (m-HCOO) and bidentate (b-HCOO), with bidentate being the favorable species. In order to form CO, HCOO dissociates into HCO (R20, Δ*E*^≠^ = 1.50 eV) and then dehydrogenates into CO (R-19, Δ*E*^≠^ = 0.06 eV). Regardless, both HCOO species have high energy barriers, making its production from CO_2_ quite difficult from an energetic point of view. On the other hand, COOH formation exhibits smaller barriers, specially assisted by OH and H_2_O. b-HCOO and COOH can dissociate into HCO (R20, Δ*E*^≠^ = 1.50 eV) and COH (R21, Δ*E*^≠^ = 1.78 eV), respectively, and then form CO through R-19 and R-17 reactions. In addition, COOH can directly dissociate into CO following R16 with a lower energy barrier than that of R21. From all these energy values, it can be stated that CO is very stable in Ru_1_@S-1, so it is expected that high temperatures will be necessary to progress into other mechanisms of the pathway. The snapshots for the stationary points (i.e., IS, TS, and FS) associated with reactive processes R9-R47 in Ru_1_@S-1 can be found in Figures S2a–h of the Supporting Information.

In the case of the formation of CH_4(g)_, the direct route is through CO dissociation (R18, Δ*E*^≠^ = 4.01 eV) and consecutive carbon hydrogenations (R22-R25, Δ*E*^≠^ = 1.37, 0.96, 0.77, and 0.63 eV, respectively). However, the direct dissociation of CO exhibits a large energy barrier, so an alternative pathway would be needed to reach C. In another study^23^ with nanoclusters of Ru over Ru (0001), methane was formed through two different routes. In the first route, CO_2_ dissociates into CO, hydrogenates sequentially HCO → CH_2_O → CH_3_O, then deoxygenates into CH_3_, and finally forms CH_4(g)_. In the system studied here, the main difficulty to follow this mechanism is that once CH_2_O is formed from HCO (R26, Δ*E*^≠^ = 0.85 eV), it is more likely to go back to HCO (R-26, Δ*E*^≠^ = 0.60 eV) than hydrogenate again into CH_3_O (R27, Δ*E*^≠^ = 1.28 eV) considering not only the barrier height but also the fact that R26 and R27 correspond to unimolecular and bimolecular processes, respectively. In addition, CH_2_O could be desorbed specially at high T and P. In the second route proposed in ref (23), COH first dissociates into C and then sequentially hydrogenates until CH_4_ is obtained. However, in Ru_1_@S-1, the reduction of COH to C (R40, Δ*E*^≠^ = 1.57 eV) exhibits a high energy barrier, so once COH is formed there are two possible reactions more favored than R40, from an energetical point of view, the conversion of COH to CO (R-17, Δ*E*^≠^ = 0.90 eV) and to COOH (R-21, Δ*E*^≠^ = 1.29 eV). HCOOH can be achieved through two different routes, by HCOH oxidation (R33, Δ*E*^≠^ = 1.35 eV) or by HCOO hydrogenation (R32, Δ*E*^≠^ = 1.39 eV and R31, Δ*E*^≠^ = 1.58 eV, from m- and b-HCOO, respectively) as in the CO_2_ hydrogenation on Pd–Mn_*x*_@S-1 conducted by Sun et al.,^11^ where HCOOH was obtained through R32.

Finally, obtaining CH_3_OH from HCOH by direct hydrogenations (R29 and R30) exhibits energy barriers of 1.00 and 1.15 eV, respectively. However, R29 is an endothermic reaction where the barrier is the full endothermicity, and thus, the backward reaction (R-29) is likely to occur, reducing the amount of CH_2_OH and, consequently, the possibility of obtaining methanol through R30. According to this, CH_3_OH could be formed by consecutive hydrogenations from CH_2_O (R27, Δ*E*^≠^ = 1.28 eV and R36, Δ*E*^≠^ = 1.41 eV).

**Figure 3:**
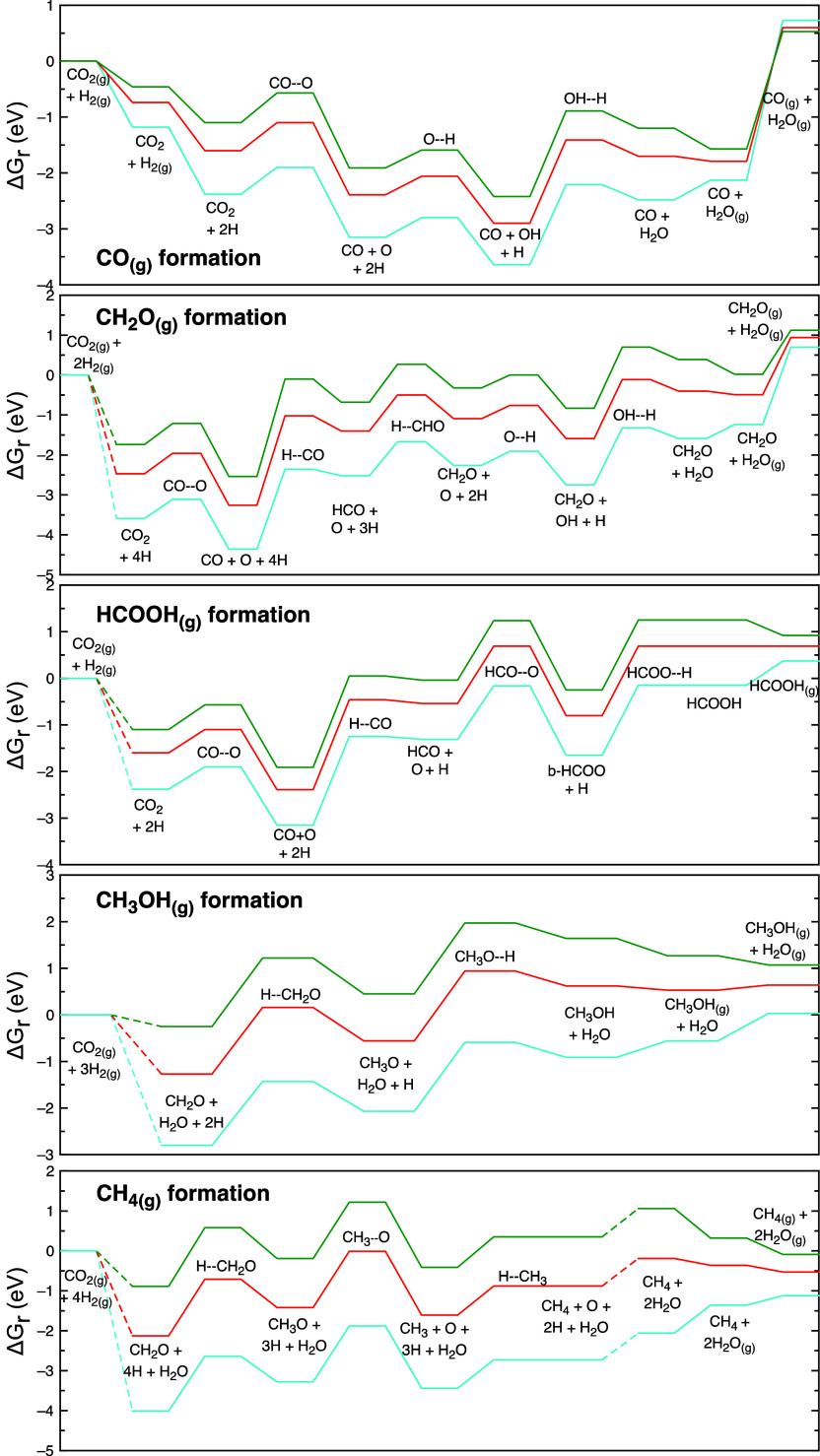
Gibbs free energy diagrams for the most favorable pathway for CO_(g)_, CH_2_O_(g)_, HCOOH_(g)_, CH_3_OH_(g)_, and CH_4(g)_ formation at 300 K (blue), 600 K (red), and 800 K (green) and 1 bar for an initial mixture of H_2_/CO_2_ in a molar ratio of 4:1. All values include the zero-point energy, and co-adsorbed species have been computed independently. Moreover, species that remain unchanged in any elementary step are not meant to participate in that step but are maintained there only to keep a proper total energy reference throughout all the steps. Dashed lines represent skipped steps for avoiding repetitiveness.

Figure 3 shows the Gibbs free energy diagrams for all gas products considered. In all of them, the most favorable pathways from an energetic point of view are exposed to several temperatures. Note that Figure 3 is constructed by addition of fragments to reproduce properly the Gibbs free energy from gas-phase reactants to the gas-phase products. Nevertheless, the data listed in Table 1 were obtained as co-adsorbed species. Gibbs reaction energy and energy barriers for adsorbed species do not have a noticeable variation as the T increase in the interval of temperature explored as there is only vibrational contribution to the internal energy and entropy, so the Gibbs free energy diagrams for these reactions are very similar to the DFT energy diagrams in Figure S3 of the Supporting Information. However, the adsorption and desorption energies have a considerable dependence on the temperature. This dependence is evidenced in Figure 3 by the increase in the adsorption Gibbs free energy values and the decrease in the Gibbs free energy in the desorption processes. This is expected since at high temperatures, gas-phase species are preferred over the adsorbed ones. As discussed before, looking at the energy barriers and reaction energies at different temperatures, the easiest method of forming CO is by direct CO_2_ dissociation (R9), whereas H_2_O formation is done by two consecutive hydrogenations over an O adatom (R44, Δ*E*^≠^ = 1.09 eV and R45, Δ*E*^≠^ = 1.38 eV). CO hydrogenates to HCO (R19, Δ*E*^≠^ = 1.99 eV) and hydrogenates again to CH_2_O (R26, Δ*E*^≠^ = 0.85 eV), and then, it can desorb or hydrogenate again until CH_4_ or CH_3_OH. HCO can also oxidize to HCOO (R-20, Δ*E*^≠^ = 0.93 eV) that can lead to HCOOH by further hydrogenation (R31, Δ*E*^≠^ = 1.58 eV).

We have just discussed the preferred pathways, according to the DFT results, to form all of the possible gas products. However, unravelling the preferred pathway is difficult by just analyzing only the DFT data or even Δ*G*(*T*) values as there are different mechanisms competing with each other and the forward and reverse reactions have a great influence in the coverage.^53^ These effects can be considered more accurately by means of the MkM simulations described below.

### 3.2 Microkinetic Modeling Results

MkM simulations based on DFT data have been performed to evaluate the catalytic activity of Ru_1_@S-1 at different temperatures (300–800 K) and pressures (1, 5, and 10 bar). The simulations provide information about the viability of the different mechanisms studied, the RDSs, and the influence of changing temperature and pressure on the TOF of the different products. The initial gas composition corresponds to a mixture of H_2_/CO_2_ in a molar ratio 4:1 as the stoichiometry of Sabatier reaction also known as methanation of CO_2_.

The first important elementary reaction is the dissociation of CO_2_ into CO and O (R9). Fortunately, at all temperatures and pressures studied, the conversion is extremely favored, producing large amounts of adsorbed CO and O. Temperature does not modify the Gibbs free energy much from the DFT energy (corrected with ZPE) of the reaction or the Gibbs barrier (i.e., Δ*G*_r_^≠^ = 0.53 eV and Δ*G*_r_ = −0.68 eV at 800 K). Increasing the pressure does not directly affect this reaction but increases the rate of adsorption (*k*_1_) of CO_2_, increasing the amount of adsorbed CO_2_, which could dissociate into CO + O.

**Figure 4:**
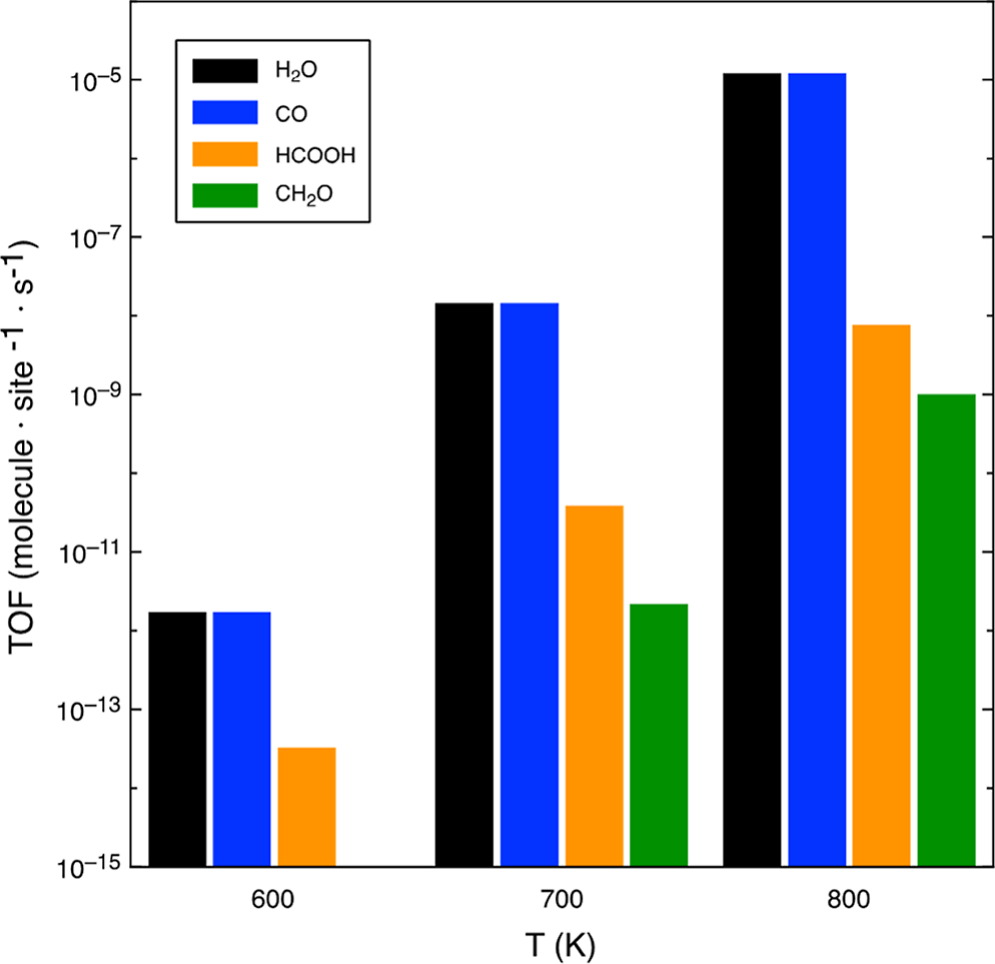
TOF for H_2_O_(g)_, CO_(g)_, HCOOH_(g)_, and CH_2_O_(g)_ products at 1 bar and temperatures in the range 600–800 K. The molar ratio of the initial mixture H_2_/CO_2_ is 4:1.

Figure 4 shows the TOF obtained for H_2_O_(g)_, CO_(g)_, CH_2_O_(g)_, and HCOOH_(g)_ at 1 bar and temperatures in the range of 600–800 K. These are the main species produced during the process, as shown in Figure S4a, which reports the TOF for all the gaseous species. As it could be inferred by DFT results, at low temperatures (<600 K), the gas production is low. The main reason for this is that adsorbed CO is very stable and reactions that can hydrogenate the CO species to produce HCO or COH (R19 and R17, respectively) exhibit high energy barriers (i.e., 1.99 and 3.08 eV, respectively). The small amount of both HCO and COH species produced does not allow further hydrogenation. Instead, HCO or COH prefers to dissociate back into CO through reactions with lower energy barriers (R-19, Δ*E*^≠^ = 0.06 eV and R-17, Δ*E*^≠^ = 0.90 eV, respectively) or generate b-HCOO, from HCO through R-20 (Δ*E*^≠^ = 0.93 eV) or COOH from COH through R-21 (Δ*E*^≠^ = 1.29 eV). In addition, the HCOO or COOH produced can dissociate into CO_2_, creating a closed system of reactions that does not produce any other gaseous product. In addition, in Figure 4, it can also be seen that the highest product obtained is H_2_O and matches stoichiometrically with the CO production at a range 1 to 1, as expected from the reaction CO_2_ + H_2_ → CO + H_2_O.

Nonetheless, at temperatures higher than 700 K, it is worth noting that the production of species like CO, CH_2_O, and HCOOH increases. This fact means that the system has enough energy to overcome not only the CO desorption barrier but also the HCO formation barriers. The initial H_2_/CO_2_ ratio of 4:1 was selected with the aim of allow the formation of CH_4(g)_, although the main species obtained during the simulations were CO and CH_2_O as well as HCOOH but in a lower extent and with only residual amounts of CH_3_OH and CH_4_. The main gas obtained being CO_(g)_ and H_2_O_(g)_ agrees with the results obtained by Lozano-Reis et al.^2^ on Ni(111) with a similar reaction pathway, even though that the desorption CO energy on Ni(111) is almost 50% smaller than the value reported in this study. The experimental study by Zieliński et al.^26^ found that Ru atoms supported on S-1 produce CO and CH_4_ at 1 atm and with the same initial H_2_/CO_2_ ratio of 4:1 used here. However, we mainly produce CO, in agreement with the results obtained in ref (23) for Ru_1_/Ru(0001). There, the selectivity to CO was reduced, increasing the number of Ru atoms in the cluster and leading to a selectivity of unity to CH_4_ for Ru_4_/Ru(0001). This could explain why in our system only CO is found and points toward the increase in the size of the Ru cluster also in S-1. Moreover, Zieliński et al.^26^ also found that the number of CO_2_ molecules converted per second increases as the Ru particle size increases, also in line with the low CO_2_ consumption obtained in this study attributed to the presence of a single Ru atom in the S-1 structure.

One can think that there is a contradiction between these productions at high temperature (Figure 4) with the Gibbs free energy diagrams in Figure 3 because at high temperatures, the adsorption of the CO_2_ and H_2_ species corresponds to endothermic processes, suggesting a less favorable reactivity pathway at high temperatures. However, this also applies for desorption processes, as for instance the CO gas production, which indicates that according to the Gibbs free energy diagram in Figure 3, desorption energy decreases substantially at high temperatures, making CO_(g)_ production more feasible than at lower temperatures. It is important to note that the increase of CO_2_ conversion with temperature agrees with the experimental results in ref (26).

**Figure 5:**
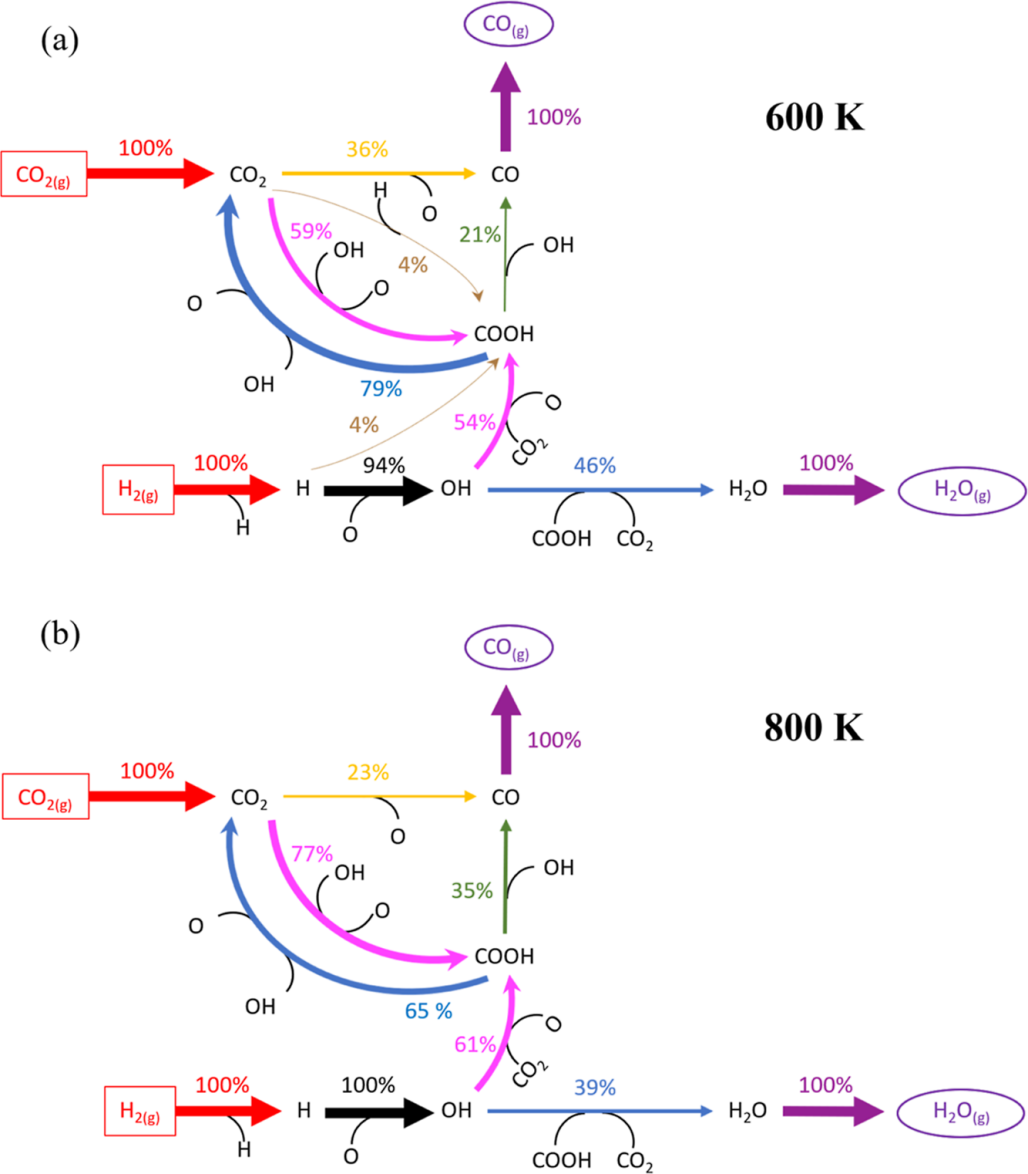
Reaction net rate pathways obtained at the MkM steady state at 1 bar and *T* = 600 K (a) and *T* = 800 K (b). Each reaction is represented by a different color. Red and purple arrows denote CO_2(g)_ and H_2(g)_ adsorptions and CO_(g)_ and H_2_O_(g)_ desorptions, respectively. Gold represents the redox mechanism (R9) and brown and pink correspond to the carboxyl formation through H (R13) and OH (R14), respectively. Dark blue denotes the dissociation of COOH assisted by OH into CO_2_ and O (R-15), dark green denotes the COOH dissociation into CO and OH (R16), and finally, black denotes the OH formation from H and O (R44). The arrow direction denotes the net flow of the elementary step, and the arrow thickness denotes the percentage of consumption of the reactant at each reaction involved. Notice that the net rate thresholds used were 10^–14^ molecules·site^–1^·s^–1^ at 600 K and 10^–7^ molecules·site^–1^·s^–1^ at 800 K, values that are 100 times lower than the highest net rates.

The MkM rates obtained for each reaction at the steady state have been analyzed following the consumption-based normalization method developed by Gupta and Vlachos^54^ to distinguish numerically and visually the most relevant reactions that contribute to the formation of the gas products. The method considers the percentage of consumption of one *X* species in the *i*-th reaction, *e*_*i*,*X*_, according to
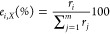
7where *r*_*i*_ is the net rate of the *i*-th reaction that is normalized by the sum of all the *m* net reaction rates implied in the *X* species consumption. The results are shown in Figure 5 at two temperatures, 600 and 800 K and 1 bar, in which only reactions with a net rate higher than 10^–14^ and 10^–7^ molecules·site^–1^·s^–1^ at 600 and 800 K, respectively, are included in the analysis to clarify and emphasize the more relevant reactions at these conditions. In Section S7, in Tables S3 and S4 (in the Supporting Information) are listed the set of rates used for constructing Figure 5. Notice also that the net rate for R15 at both temperatures is negative; therefore, R-15 is used in Figure 5 instead of R15.

**Figure 6:**
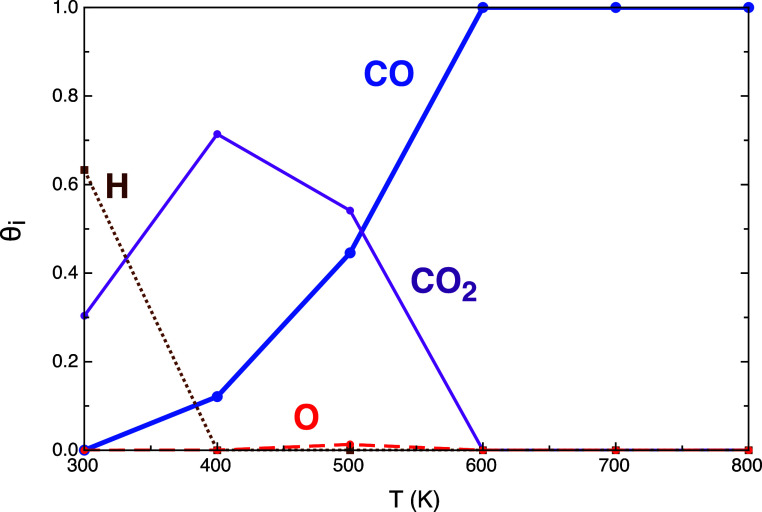
Surface coverage of the main adsorbed species (CO_2_, CO, H, and O) at 1 bar and temperatures in the range of 300–800 K. The molar ratio of the initial mixture H_2_/CO_2_ is 4:1.

As expected from the DFT energy barriers and the data shown in Figure 3, the CO could be produced through the redox mechanism or, equivalently, through the direct dissociation of CO_2_ (R9). However, the MkM simulations found that CO is also formed through the COOH route assisted by OH (R14) too, and then the COOH dissociates into CO + OH (R16). In Figure 5a,b, it can be noticed that the consumption of CO_2_ into COOH increases with the temperature from 59% to 77% but decreases for the redox path from 36% to 23%. Another important result is that H_2_O is produced by R-15, which converts OH and COOH into CO_2_ and H_2_O instead of the direct association of OH with H (R45) or OH (R46). Main part of CO is desorbed to form CO_(g)_ (R3), but there is a small net rate for the HCO production through R19, despite the high energy barrier associated with the reaction. This can be explained by the high amount of CO coverage present in the system (θ_CO*_ ≃ 1 at temperatures higher than 600 K, as shown in Figure 6), so despite the high energy barrier associated, there is still a net positive reaction rate for R19. The HCO species opens three main routes: first, the formation of b-HCOO (R-20) that can be further hydrogenated to HCOOH (R31) and then desorbed (R4) and, second, the formation of CH_2_O (R26). Both routes are the same reaction pathways predicted for HCOOH_(g)_ and CH_2_O_(g)_ production in Figure 3. Third, HCOH (R34) can dissociate into COH (R-28) that can be subsequently reduced into C (R40) to finally form CO (R-18). In the case of CH_2_O, practically all the species formed desorb to the gas phase (R7), making the production of CH_3_OH_(g)_ and CH_4(g)_ almost negligible compared to that of CO_(g)_, HCOOH_(g)_, and CH_2_O_(g)_. H_2_O is mainly produced by R45. With all these results presented, we can conclude that our Gibbs free energy diagram (Figure 3) and the reaction pathway obtained with MkM simulations (Figure 5) agree, but the MkM analysis provides a richer understanding of all the possible pathways for the gas production.

The DRC analysis was done for the four main gas products formed (i.e., H_2_O_(g)_, CO_(g)_, CH_2_O_(g)_, and HCOOH_(g)_). The MkM results showed that the RDS for the CO_(g)_ and H_2_O_(g)_ production was clearly the carbon monoxide desorption (R3) as we expected due to the high barrier associated with the process. This could explain why at higher temperatures, the TOF for CO_(g)_ increases substantially, as shown in Figure 4. At high temperatures, Δ*G*^≠^ decreases substantially, allowing the high coverage of CO presented in the system to desorb. Similarly, in the case of CH_2_O, the DRC analysis showed that the RDS also has its own desorption process (R7).

Changing pressures does not change significantly the gas production ratios between CO, CH_2_O, and HCOOH, as can be seen in Figure S4b. However, the TOF increases for all gaseous products as the pressure increases and also by increasing the adsorption rates of both reactants, CO_2_ and H_2_. Besides, the H_2_/CO_2_ initial composition was modified, although no significant changes were found in the TOF and in the CO, O, and H coverages.

Finally, the apparent activation energies (*E*_*a*_) for the formation of CO_(g)_, CH_2_O_(g)_, and HCOOH_(g)_ products were obtained from eq 6 through a *ln r*_*i*_*vs* 1000·*T*^–1^ fitting within the range of 600–800 K (see Table 2 and Section S8, Figure S5 and Table S4—in the Supporting Information). The *E*_a_ values for CO_(g)_ and CH_2_O_(g)_ are very similar to their desorption energy barriers, remarking the results found in the DRC analysis. However, in the case of HCOOH_(g)_ formation, there is no energy barrier in its pathway that corresponds with the *E*_a_ value found.

**Table 2:** Pre-exponential Factor, *A*_*i*_, and Apparent Activation Energies, *E*_a_, for the CO_(g)_, HCOOH_(g)_, and CH_2_O_(g)_ Production at 600–800 K Obtained from Fitted Parameters Shown in Section S8 in Supporting Information

gas produced	*A*_i_/s^–1^	*E*_a_/eV
CO_(g)_	4.32 × 10^15^	3.26
HCOOH_(g)_	9.97 × 10^7^	2.56
CH_2_O_(g)_	6.33 × 10^9^	2.98

## 4 Conclusions

In our previous work,^27,28^ we studied the CO_2_ activation for several TMs that presented good stability on the S-1 like Ru. Now we are computing a full reaction pathway (47 reversible reactions) with several gas products, allowing the system to form all possible gas products studied (e.g., CH_4(g)_) and not favoring any of them. In addition, complete and detailed MkM simulations were performed to check the gas production at different temperatures and pressures, including DRC analysis. CO_2_ hydrogenation on Ru@S-1 was studied by DFT calculations and microkinetic simulations. It has been shown that at low temperatures, no gaseous production is observed due to the high energy barriers associated with the carbon monoxide desorption as well as to the COH and HCO formation reactions from CO, which avoid the hydrogenation of species into other products. However, on increasing the temperature, the gaseous production increases, mainly to CO_(g)_ and H_2_O_(g)_, as reported by Lozano-Reis et al.^2^ on Ni(111), and also to HCOOH_(g)_ and CH_2_O_(g)_ to a lower extent. Nevertheless, the production is small due to the high CO coverage (99%). MkM provided information on the different reaction rates, showing that CO formation is produced mainly by CO_2_ direct dissociation as well as assisted by OH to form COOH that finally dehydroxylates. DRC analysis showed that the RDS for CO_(g)_ and H_2_O_(g)_ productions is CO desorption. In a similar way, CH_2_O_(g)_ production RDS corresponds to its own desorption. However, HCOOH_(g)_ production proved to be temperature dependent, mainly by HCOOH formation from b-HCOO and CO desorption.

The present study demonstrates that Ru SACs are selective toward carbon monoxide formation in the catalytic CO_2_ hydrogenation, as concluded by Ma and Wang^23^ in their previous work, where the catalyst was a single Ru atom supported on a Ru surface (i.e., Ru_1_/Ru(0001)). However, it is highly probable, as suggested by Ma and Wang^23^ and also by Zieliński et al.,^26^ that increasing the number of atoms in the Ru cluster enhances the selectivity toward methane formation. Therefore, the next step is to analyze a larger Ru atom cluster to determine the effect of cluster size in the CO_(g)_/CH_4(g)_ formation ratio. This would be the starting point of an even more ambitious project to study the catalytic properties of small clusters of Ru and other TMs inside the S-1.

## Data Availability

Optimized structures (i.e., VASP CONTCAR files) of all relevant structures have been also made available into a public GitHub repository: https://github.com/ManuelCanovas/CO2-Hydrogenation-on-Ru-encapsulated-on-Silicalite.

## Supporting Information Available

Information about how to compute rate constants, geometries of all the minima and transition states, potential energy diagrams, the TOF dependence over temperature and pressure, rates and net rates of reaction at the steady state, and the Arrhenius parameters (PDF)
