# Supplementary material for: CO2 Hydrogenation on Ru Single-Atom Catalyst Encapsulated in Silicalite: a DFT and Microkinetic Modeling Study
Source: J Phys Chem C Nanomater Interfaces. 2024 Sep 23;128(39):16551–62. doi: 10.1021/acs.jpcc.4c05941 (PMC11459948; doi:10.1021/acs.jpcc.4c05941)
Supplement: Supplementary file 1 — jp4c05941_si_001.pdf [file jp4c05941_si_001.pdf]

Supporting Information

CO_2 Hydrogenation on Ru Single-Atom Catalyst Encapsulated in Silicalite: a DFT and Microkinetic Modeling Study

Manuel A. Cánovas[1], Alejandro Gracia[1], Ramón Sayós[1], Pablo Gamallo[1,*]

[1] Departament de Ciència de Materials i Química Física & Institut de Química Teòrica i Computacional (IQTCUB), Universitat de Barcelona, C. Martí i Franquès, 1, 08028, Barcelona, Spain.

*corresponding author: gamallo@ub.edu

S1. Computing rate constants S2
S2. Gas-phase and adsorbed optimal geometries for reactants and products S4
S3. Geometries of adsorbed gas species S5
S4. Geometries of reactants, transition states and products S6
S5. Potential energy diagrams S14
S6. TOF dependence over temperature and pressure S15
S7. Rates and net rates of reaction S16
S8. Arrhenius parameters S18

S1. Computing rate constants

The parameters for the microkinetic model are based on DFT data. In the present study, the adsorption processes of gas species are non-activated and thus, the rate constant for these processes ($k_{ads,i}$) have been obtained according to the Hertz-Knudsen expression,

$$k_{ads,i} = S\ \frac{P_i\, A_s}{\sqrt{2\pi m_i k_b T}} \tag{1}$$

where S is the sticking coefficient that was set to 1, P_i and m_i are the partial pressure and molecular mass of i-species, respectively, k_b is the Boltzmann constant, T is the temperature and A_s is the surface area of the active site that it was approximated as the hemisphere around the Ru atom (*i.e.,* A_s=$2\pi r_{Ru}^2 \approx 1.13e^{-19}$ m^2).

The desorption rate constants ($k_{des,i}$) have been calculated through,

$$k_{des,i} = \frac{k_b T}{h} \frac{q_{vib,g}\ q_{rot,g} q_{trans2D,g}}{q_{vib,ads}}\ e^{\frac{\Delta E_{ads,i}}{k_b T}} \tag{2}$$

where $q_{vib,g}$, $q_{rot,g}$ and $q_{trans2D,g}$ are the vibrational, rotational and translational partition functions of the gas-phase species, $q_{vib,ads}$, is the vibrational partition function for the adsorbed species, h is the Plank's constant and $\Delta E_{ads,i}$ is the adsorption energy for i-species computed through eq 1 on the main text. Finally, for the activated conversion of the adsorbed reactants (*i.e.,* Langmuir-Hinshelwood reaction: $X + Y \rightarrow Z + \ *$), the conventional transition state theory (TST) rate constant equation (k_{TST}) was used,

$$k_{LH} = \frac{k_b T}{h} A_s \frac{q_{vib}^{\neq}}{q_{vib,X} \bullet q_{vib,Y}}\ e^{-\frac{\Delta E^{\neq}}{k_b T}} \tag{3}$$

where $\Delta E^{\neq}$ is the zero-point energy corrected reaction energy barrier computed through eq. (2) and $q_{vib}^{\neq}$, $q_{vib,X}$ and $q_{vib,Y}$ are the vibrational partition function for the TS and reactants, respectively. Notice that the k_{LH} has units of m^2/s. However, as the MKMCXX program solves the differential equation for the fractional coverage of every single specie $\frac{d\theta_i}{dt}$, the unit of all rate constant must be s^{-1}. Therefore, dividing by A_s is needed to ensure correct dimensional usage. So, the implemented rate constant in the program is $k'_{LH} = \frac{k_{LH}}{A_s}$. One can check that for a j-reaction studied, the ΔG_j can be also computed as

$$\Delta G_j = -\, k_b T\, ln\left[\frac{k_{for,j}}{k_{back,j}}\right] \tag{4}$$

where $k_{for,j}$ and $k_{back,j}$ are the forward and backward rate constants for j-reaction, respectively, each one computed using eqs. (1-3) on the SI depending on the reaction type.

Besides, in the case of reactions involving a hydrogen atom transfer, their rate constants have been corrected by a semiclassical Eckart tunneling correction factor[1],

$$\kappa(T) = 1 + \frac{1}{24}\left|\frac{hv_{img}}{k_b T}\right|^2\left(1 + \frac{k_b T}{\Delta E^{\neq}}\right) \tag{5}$$

where v_{img} corresponds to the imaginary frequency associated to the TS, whereas the other variables have been defined previously.

S2. Gas-phase and adsorbed optimal geometries for reactants and products

Table S1. Optimal geometries for all the stable species in gas-phase and adsorbed on Ru_1@S-1. In addition, the bond length with Ru and the bond angle are included for the adsorbed species. All distances are in Å and angles in degrees.

Species	Bond length	Gas-Phase	Adsorbed	Bond length		Bond angle	
CO_2	*d*(C-O)	1.17	1.28	*d*(Ru-C)	1.98	O-C-O	141
				d(Ru-O)	2.11		
H_2	*d*(H-H)	0.75	2.02	*d*(Ru-H)	1.57		
CO	*d*(C-O)	1.14	1.18	*d*(Ru-C)	1.81		
HCOOH	*d*(C-H)	1.10	1.10	*d*(Ru-O)	2.00	O-C-H	120
	d(C-O)	1.36	1.34	*d*(Ru-C)	3.01	O-C-O	121
	d(C-O)	1.21	1.24			C-O-H	110
CH_3OH	*d*(C-O)	1.43	1.45	*d*(Ru-O)	2.12	O-C-H	108
	d(C-H)	1.10	1.10	*d*(Ru-C)	3.15		
	d(O-H)	0.97	0.97				
CH_4	*d*(C-H)	1.10	1.20	*d*(Ru-C)	2.17	H-C-H	103
CH_2O	*d*(C-O)	1.21	1.35	*d*(Ru-C)	2.03	O-C-H	117
	d(C-H)	1.11	1.10	*d*(Ru-O)	1.96	H-C-H	115
H_2O	*d*(O-H)	0.97	0.98	*d*(Ru-O)	2.17	H-O-H	106

S3. Geometries of adsorbed gas species

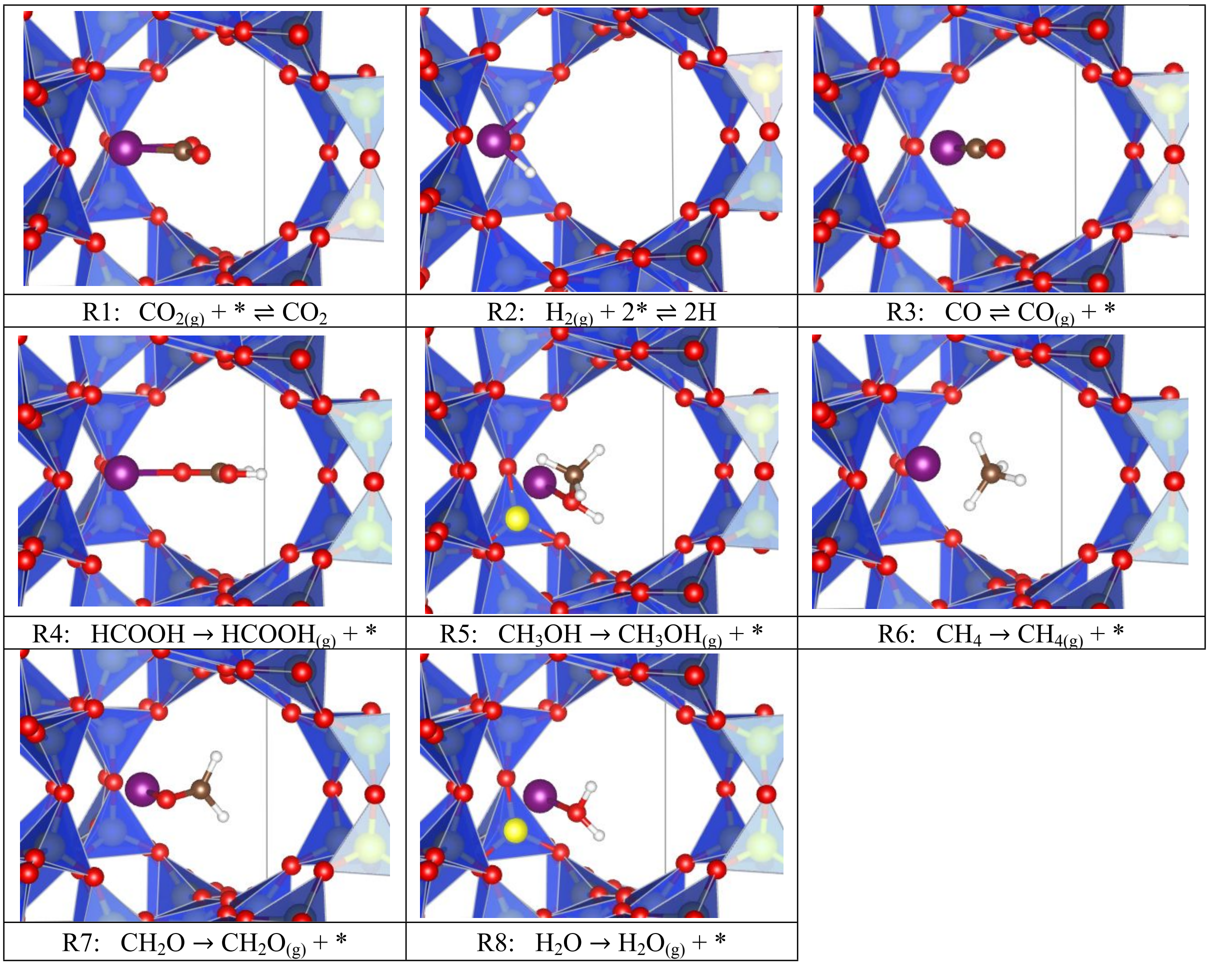

Figure S1. Snapshots with the configurations of the adsorbed gases studied in Ru_1@S-1.

S4. Geometries of reactants, transition states and products

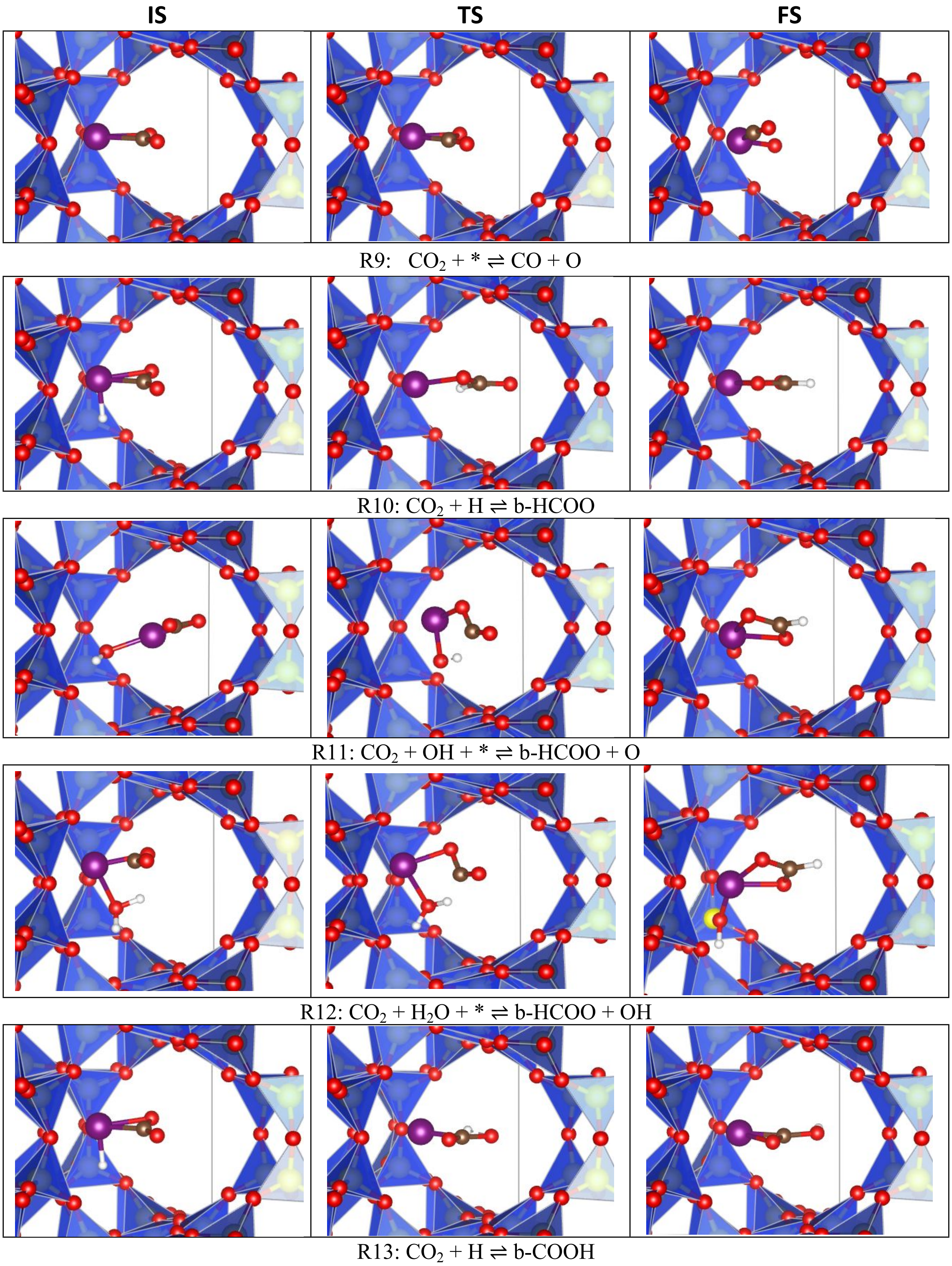

Figure S2a. Snapshots with the configurations of the reactants (IS, left), transition state (TS, middle) and products (FS, right) for R9 – R13 reactions on Ru_1@S-1. The reactions correspond to the same notation used in Table 2 and Figure 2.

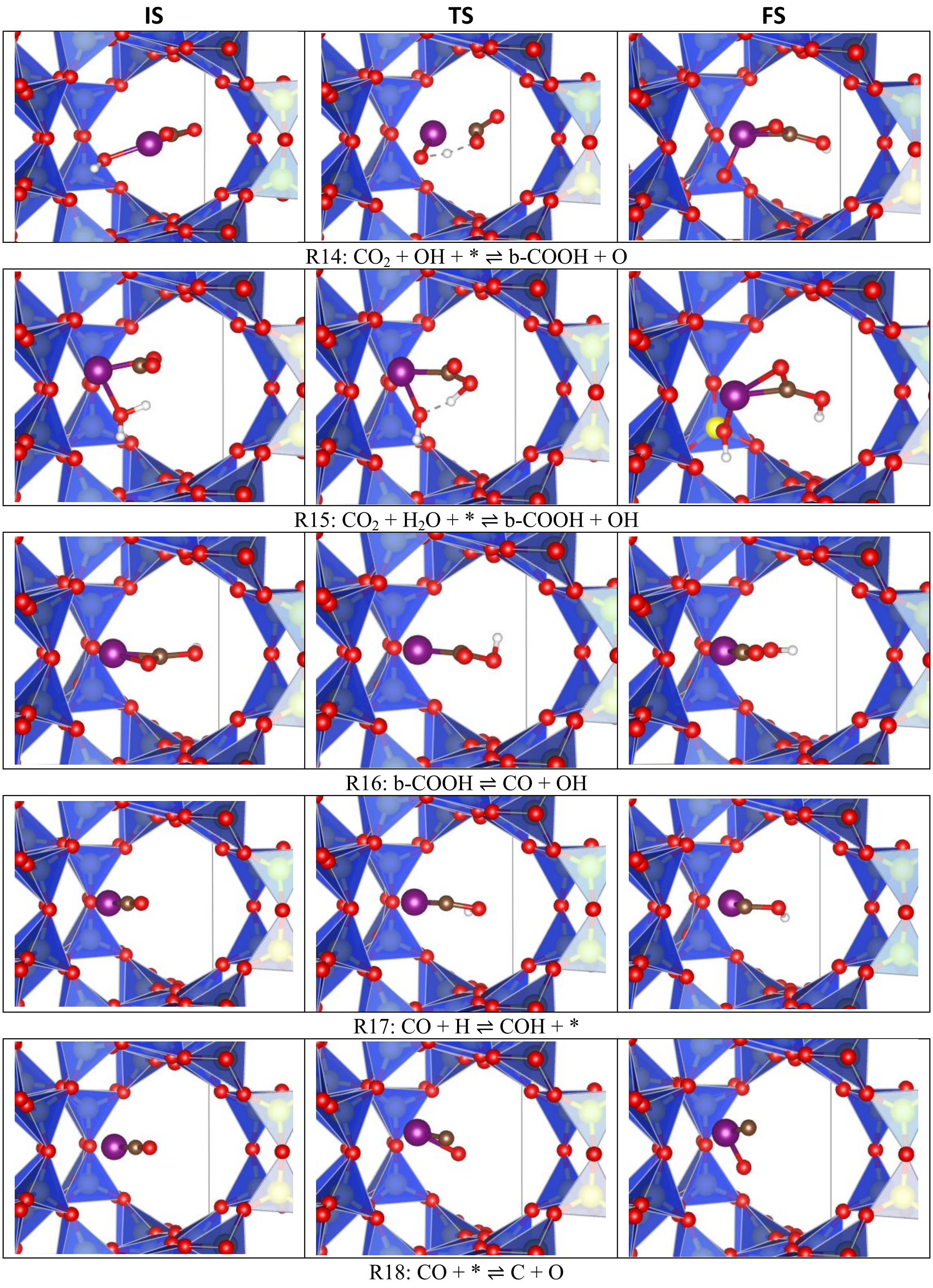

Figure S2b. Snapshots with the configurations of the reactants (IS, left), transition state (TS, middle) and products (FS, right) for R14 – R18 reactions on Ru_1@S-1. The reactions correspond to the same notation used in Table 2 and Figure 2.

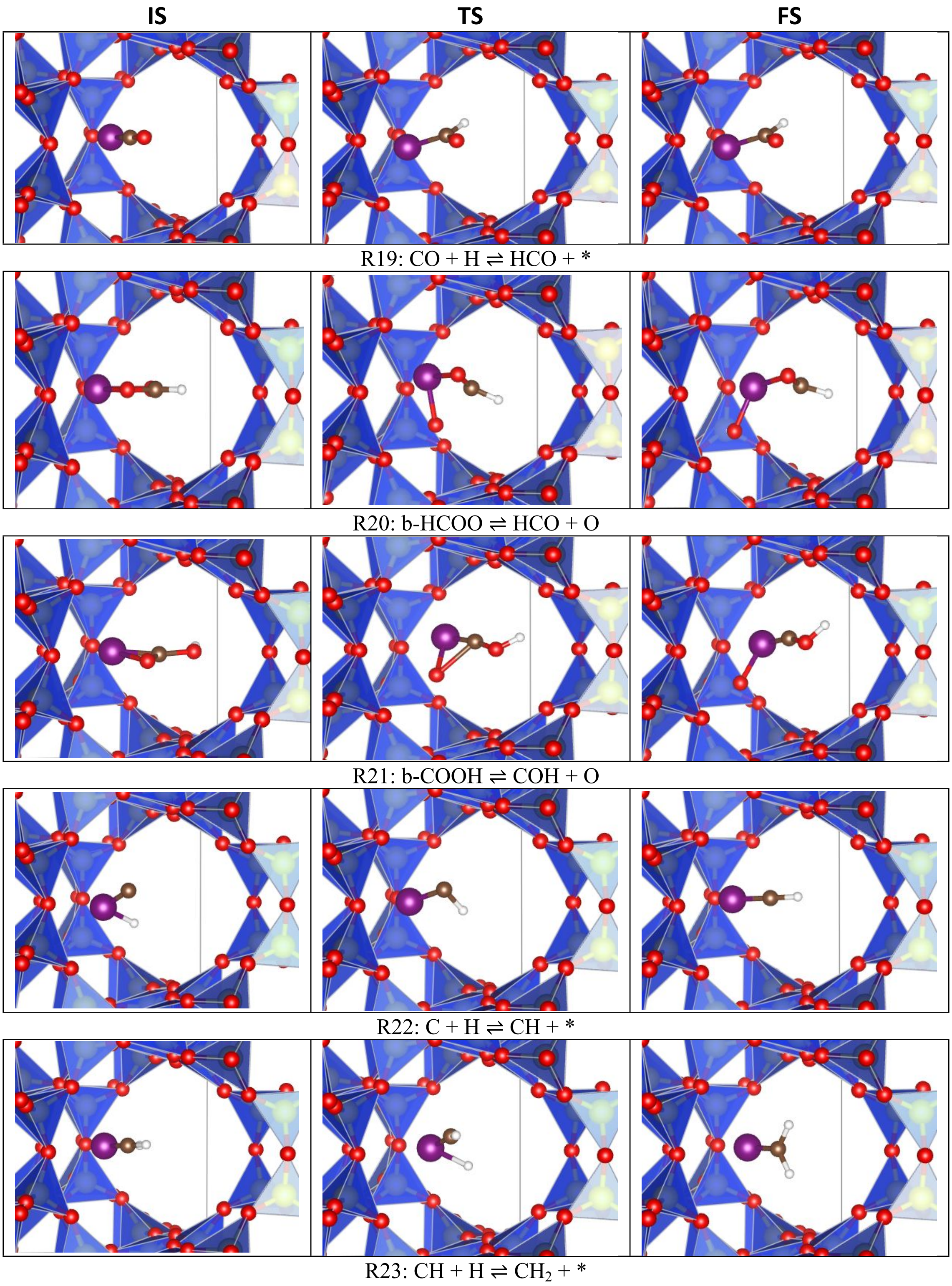

Figure S2c. Snapshots with the configurations of the reactants (IS, left), transition state (TS, middle) and products (FS, right) for R19 – R23 reactions on Ru_1@S-1. The reactions correspond to the same notation used in Table 2 and Figure 2.

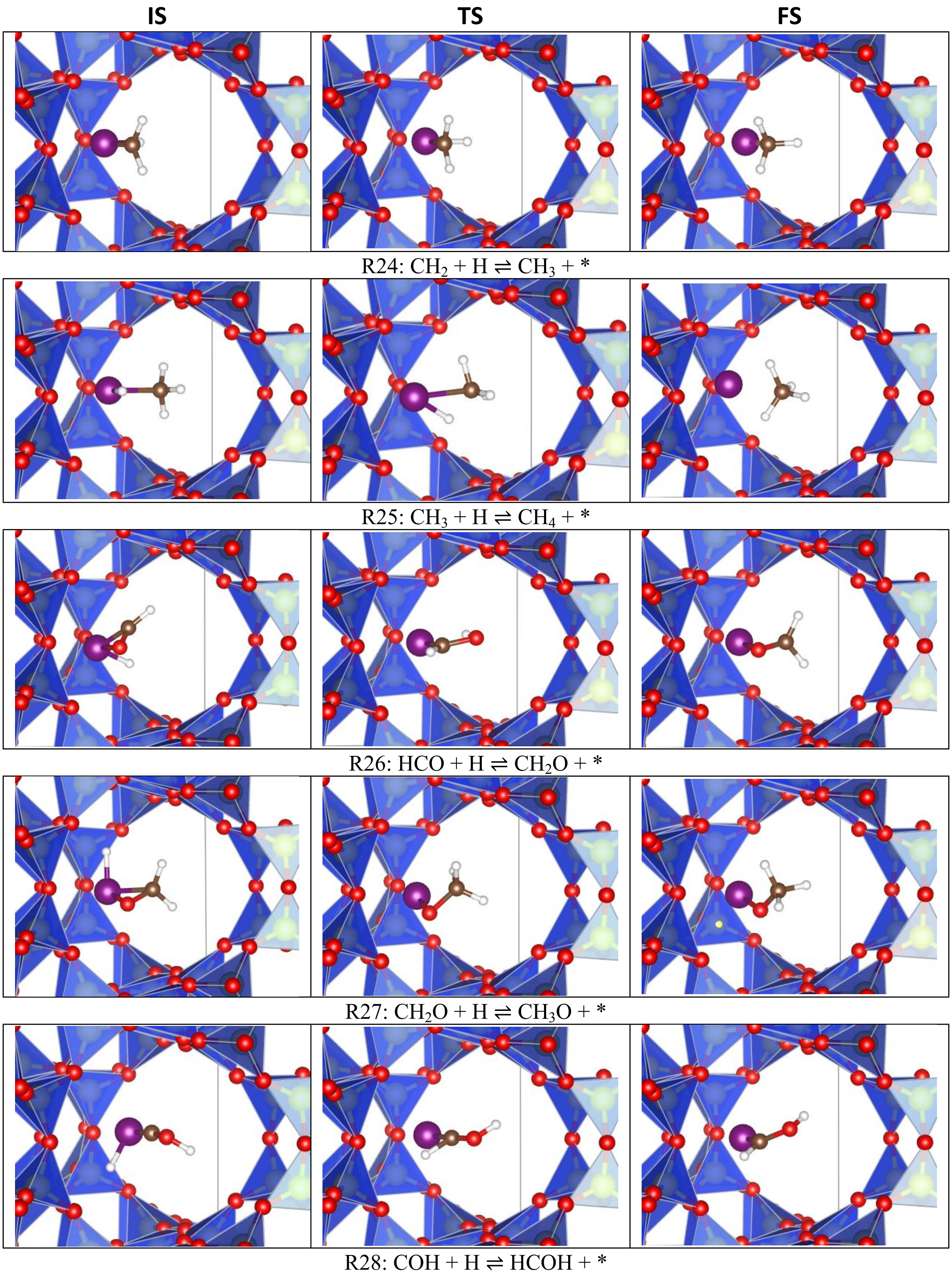

Figure S2d. Snapshots with the configurations of the reactants (IS, left), transition state (TS, middle) and products (FS, right) for R24 – R28 reactions on Ru_1@S-1. The reactions correspond to the same notation used in Table 2 and Figure 2.

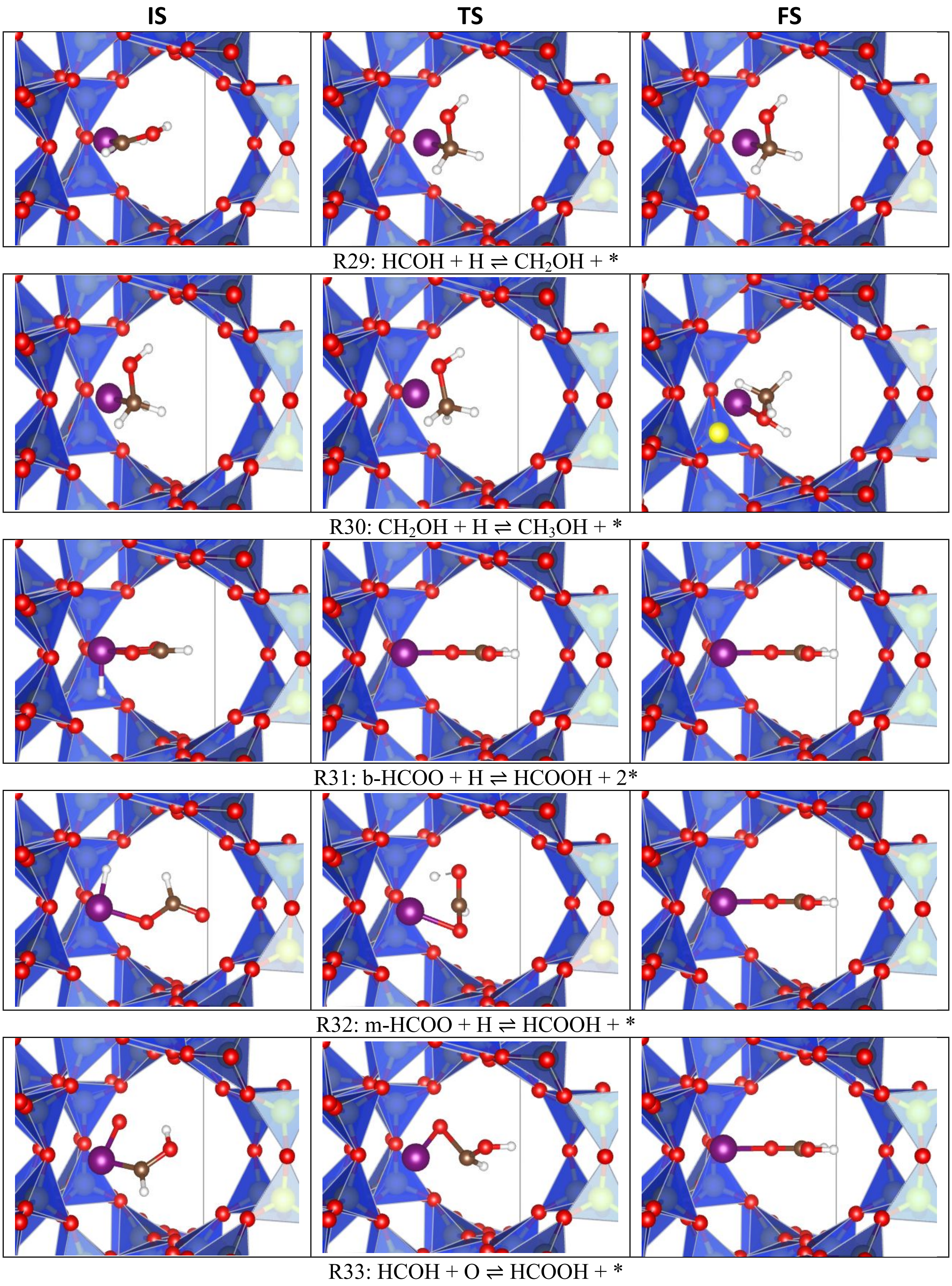

Figure S2e. Snapshots with the configurations of the reactants (IS, left), transition state (TS, middle) and products (FS, right) for R29 – R33 reactions on Ru_1@S-1. The reactions correspond to the same notation used in Table 2 and Figure 2.

IS **TS** **FS**

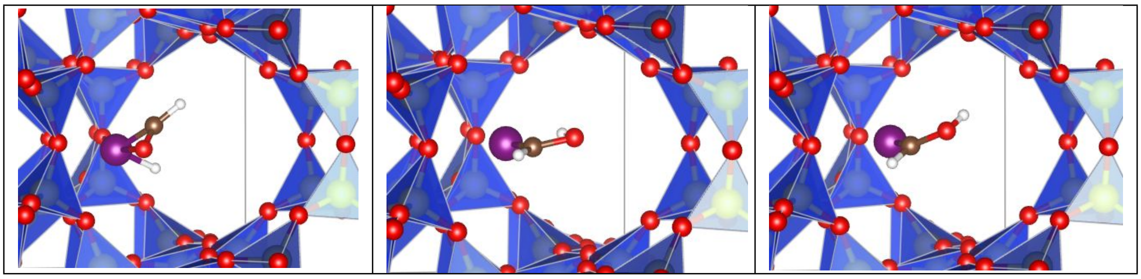

R34: HCO + H ⇌ HCOH + *

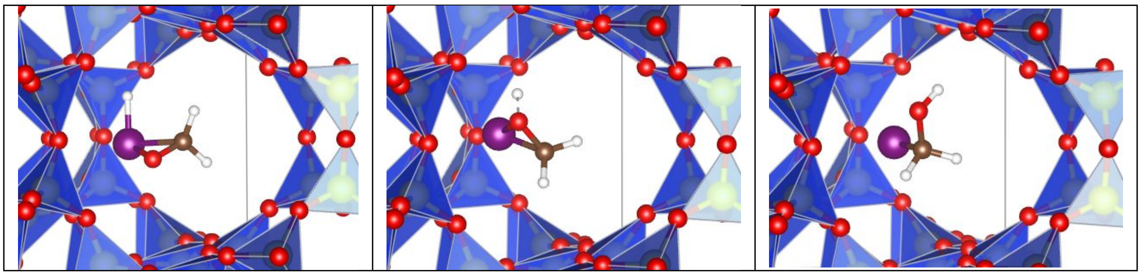

R35: CH_2O + H ⇌ CH_2OH + *

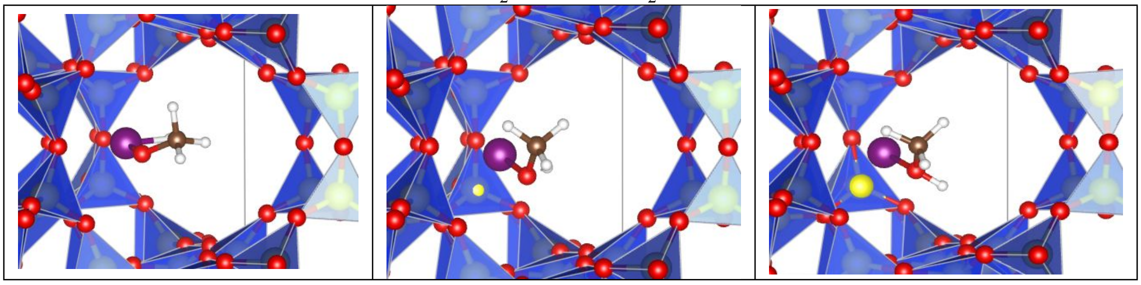

R36: CH_3O + H ⇌ CH_3OH + *

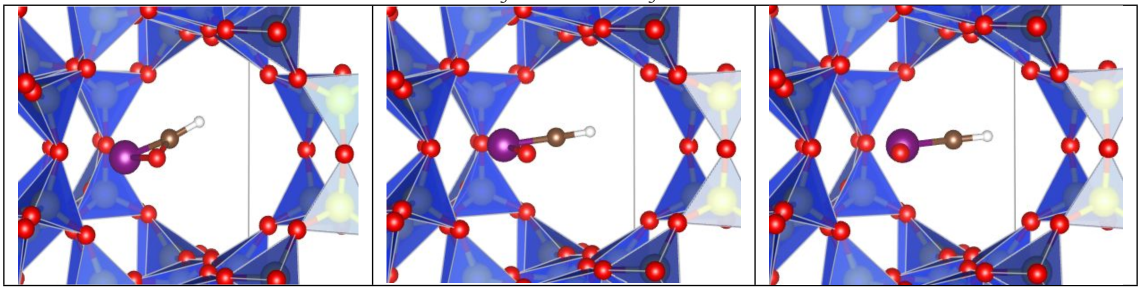

R37: HCO + * ⇌ CH + O

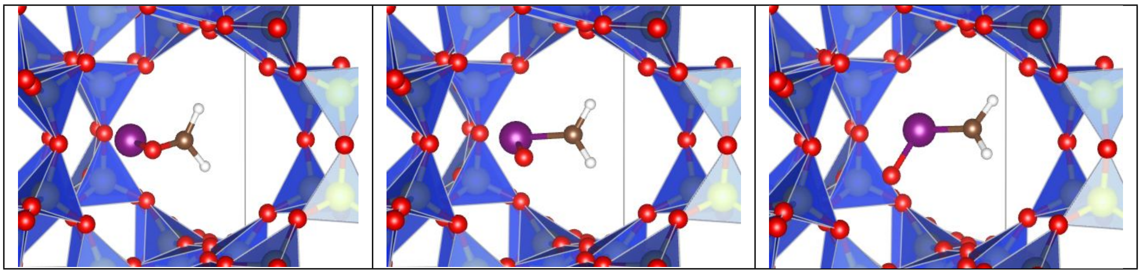

R38: CH_2O + * ⇌ CH_2 + O

Figure S2f. Snapshots with the configurations of the reactants (IS, left), transition state (TS, middle) and products (FS, right) for R34 – R38 reactions on Ru_1@S-1. The reactions correspond to the same notation used in Table 2 and Figure 2.

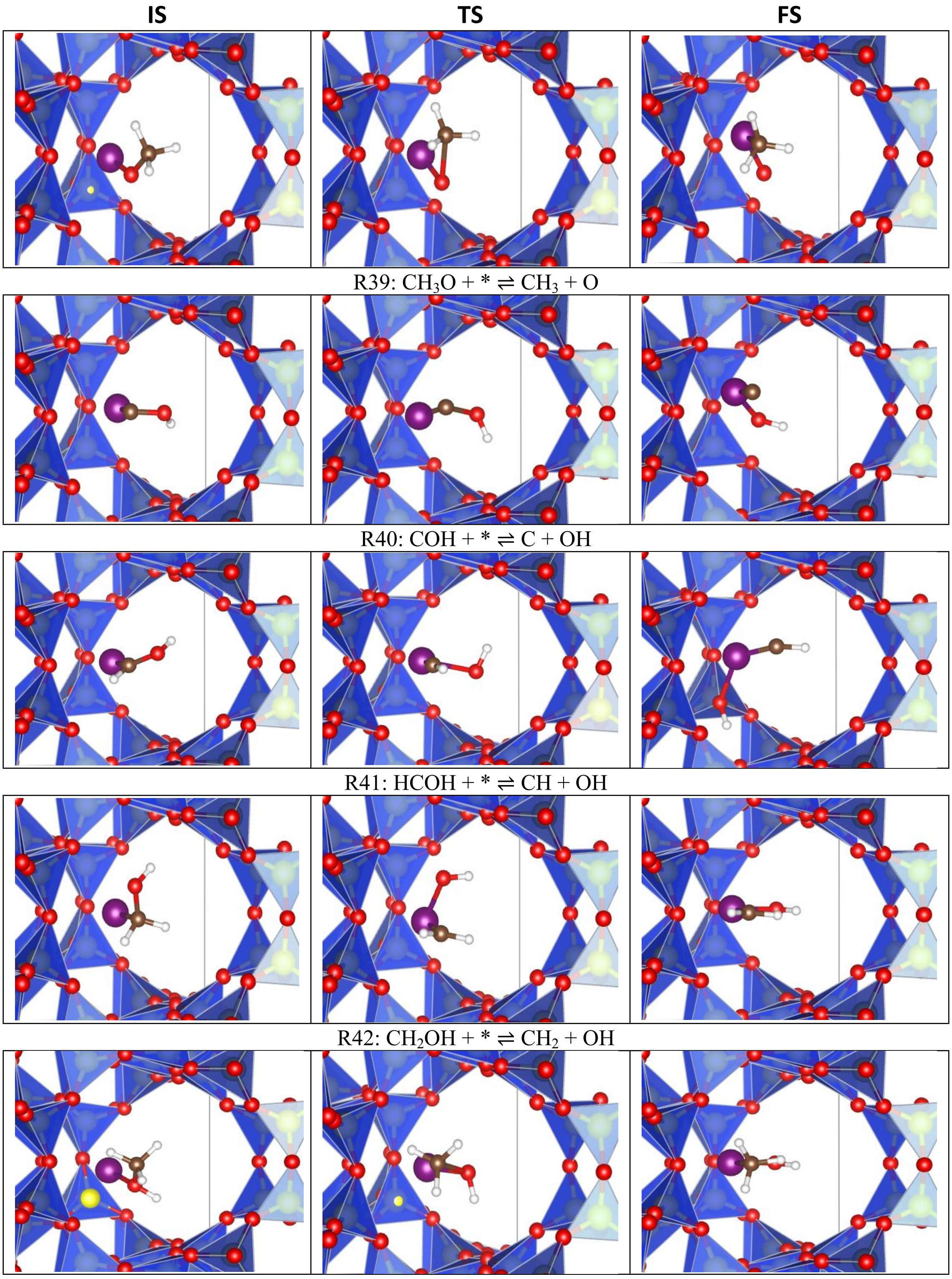

Figure S2g. Snapshots with the configurations of the reactants (IS, left), transition state (TS, middle) and products (FS, right) for R39 – R43 reactions on Ru_1@S-1. The reactions correspond to the same notation used in Table 2 and Figure 2.

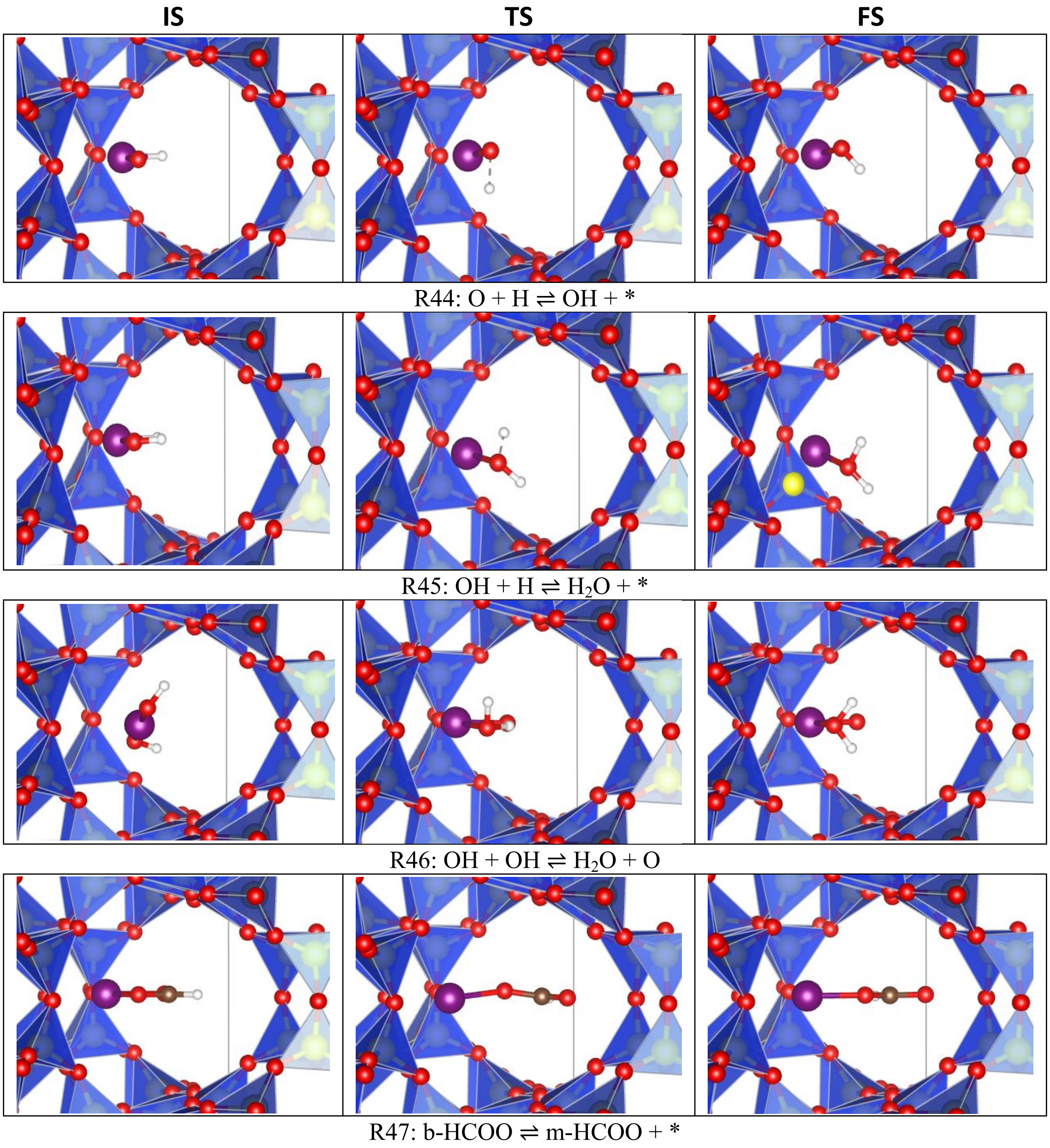

Figure S2h. Snapshots with the configurations of the reactants (IS, left), transition state (TS, middle) and products (FS, right) for R44 – R47 reactions on Ru_1@S-1. The reactions correspond to the same notation used in Table 2 and Figure 2.

S5. Potential energy diagrams

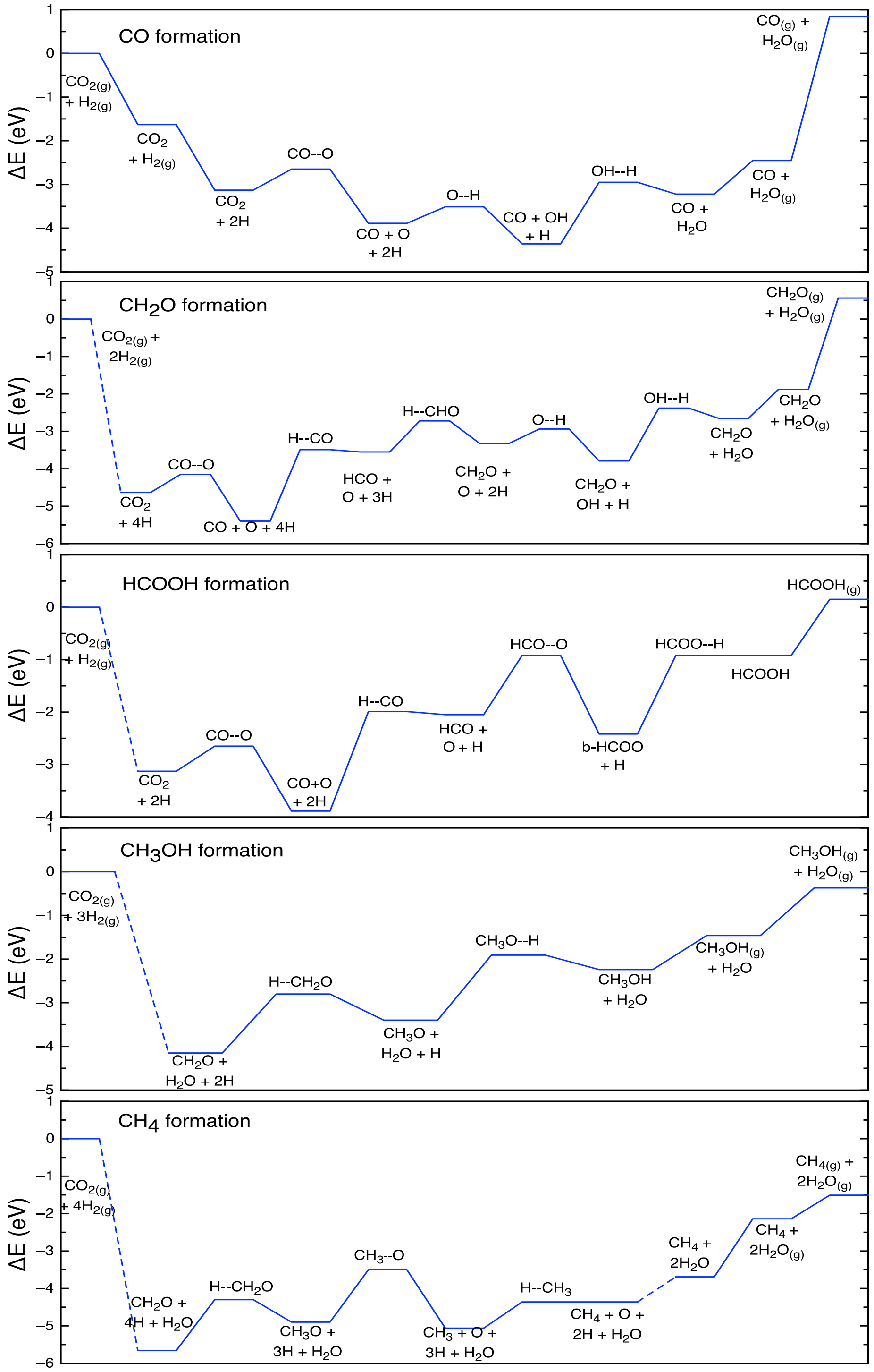

Figure S3. Potential energy diagrams for the most favourable pathway for $CO_{(g)}$, $HCOOH_{(g)}$, $CH_2O_{(g)}$, $CH_3OH_{(g)}$ and $CH_{4(g)}$ formation for an initial mixture of H_2/CO_2 in a ratio of 4:1. All values include the zero-point energy and co-adsorbed species have been computed independently. Moreover, species that remain unchanged in any elementary step are not meant to participate in that step but are maintained there only to keep a proper total energy reference throughout all the steps. Dashed lines represent skipped steps for avoiding repetitiveness.

S6. TOF dependence over temperature and pressure

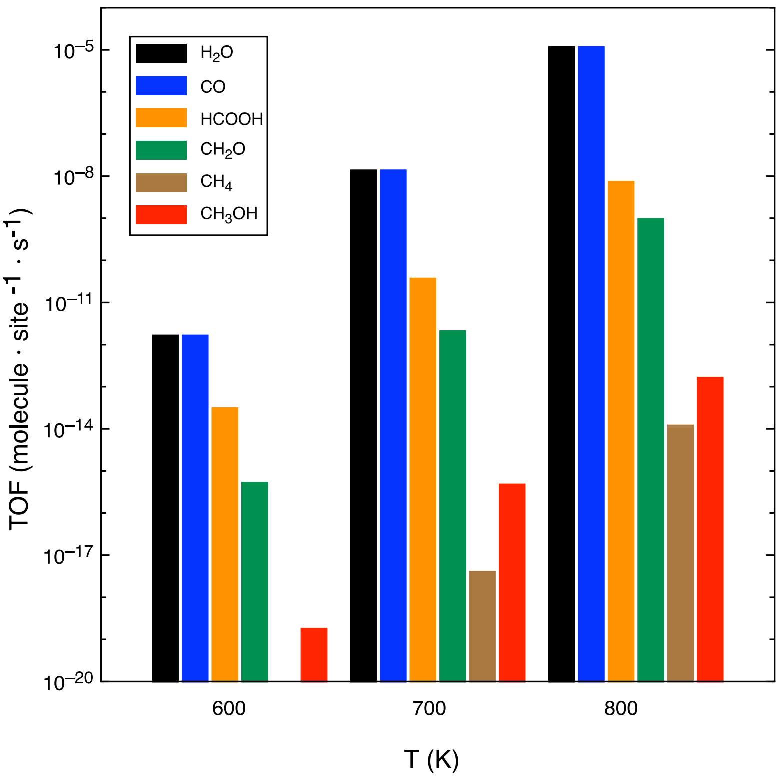

Figure S4a. TOF for all the gaseous species produced at 1 bar and temperatures in the range 600 K – 800 K. The molar ratio of the initial mixture H_2/CO_2 is 4:1.

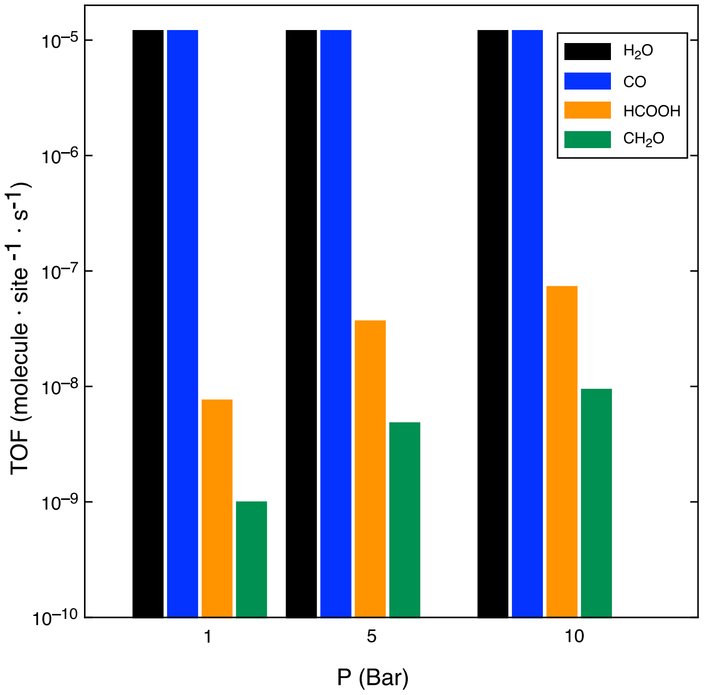

Figure S4b. TOF for H_2O, CO, CH_2O and HCOOH at 800 K and pressures of 1, 5 and 10 bar. The molar ratio of the initial mixture H_2/CO_2 is 4:1.

S7. Rates and net rates of reaction

Table S2. Forward, backward and net rates obtained from MkM simulations at T = 600 K and 1 bar, once reached the steady state. All rates have units of molecules·site^{-1}·s^{-1} and only the reactions with a net rate higher than 10^{-14} molecules·site^{-1}·s^{-1} are detailed. It is worth noting that reactions R4, R10 and R31 have not been included in Figure 5(a) as the consumption rate of H and CO_2 in R10 is lower than 1%.

Reaction	Forward rate	Backward Rate	Net rate
R1: $CO_{2(g)} + * \rightleftharpoons CO_2$	3.19×10^{-3}	3.19×10^{-3}	1.80×10^{-12}
R2: $H_{2(g)} + 2* \rightleftharpoons 2H$	5.12×10^{-12}	3.35×10^{-12}	1.77×10^{-12}
R3: $CO \rightleftharpoons CO_{(g)} + *$	1.73×10^{-12}	0.00	1.73×10^{-12}
R4: $HCOOH \rightarrow HCOOH_{(g)} + *$	3.27×10^{-14}	0.00	3.27×10^{-14}
R8: $H_2O \rightarrow H_2O_{(g)} + *$	1.73×10^{-12}	0.00	1.73×10^{-12}
R9: $CO_2 + * \rightleftharpoons CO + O$	1.66×10^{-6}	1.66×10^{-6}	1.26×10^{-12}
R10: $CO_2 + H \rightleftharpoons$ b-HCOO	1.00×10^{-10}	1.00×10^{-10}	3.27×10^{-14}
R13: $CO_2 + H \rightleftharpoons$ b-COOH	1.50×10^{-13}	2.02×10^{-15}	1.48×10^{-13}
R14: $CO_2 + OH + * \rightleftharpoons$ b-COOH + O	6.93×10^{-11}	6.73×10^{-11}	2.06×10^{-12}
R15: $CO_2 + H_2O + * \rightleftharpoons$ b-COOH + OH	6.88×10^{-17}	1.73×10^{-12}	-1.73×10^{-12}
R16: b-COOH $\rightleftharpoons$ CO + OH	4.72×10^{-13}	1.41×10^{-19}	4.72×10^{-13}
R31: b-HCOO + H $\rightleftharpoons$ HCOOH + 2*	3.13×10^{-14}	2.07×10^{-19}	3.13×10^{-14}
R44: O + H $\rightleftharpoons$ OH + *	3.32×10^{-12}	6.22×10^{-16}	3.32×10^{-12}

Notice that the net rate for R15 at both temperatures is negative. Hence, R-15 is used in Figure 5 instead of R15.

Table S3. Forward, backward and net rates obtained from MkM simulations at T = 800 K and 1 bar, once reached the steady state. All rates have units of molecules·site^{-1}·s^{-1} and only the reactions with a net rate higher than 10^{-7} molecules·site^{-1}·s^{-1} are detailed.

Reaction	Forward rate	Backward rate	Net rate
R1: $CO_{2(g)} + * \rightleftharpoons CO_2$	$1.03\times10^{+1}$	$1.03\times10^{+1}$	1.22×10^{-5}
R2: $H_{2(g)} + 2* \rightleftharpoons 2H$	6.20×10^{-5}	4.97×10^{-5}	1.22×10^{-5}
R3: $CO \rightleftharpoons CO_{(g)} + *$	1.22×10^{-5}	0.00	1.22×10^{-5}
R8: $H_2O \rightarrow H_2O_{(g)} + *$	1.22×10^{-5}	0.00	1.22×10^{-5}
R9: $CO_2 + * \rightleftharpoons CO + O$	1.36×10^{-1}	1.36×10^{-1}	5.60×10^{-6}
R14: $CO_2 + OH + * \rightleftharpoons$ b-COOH + O	4.54×10^{-5}	2.66×10^{-5}	1.88×10^{-5}
R15: $CO_2 + H_2O + * \rightleftharpoons$ b-COOH + OH	2.58×10^{-10}	1.22×10^{-5}	-1.22×10^{-5}
R16: b-COOH $\rightleftharpoons$ CO + OH	6.60×10^{-6}	1.72×10^{-10}	6.60×10^{-6}
R44: O + H $\rightleftharpoons$ OH + *	2.54×10^{-5}	1.09×10^{-6}	2.44×10^{-5}

Notice that the net rate for R15 at both temperatures is negative. Hence, R-15 is used in Figure 5 instead of R15.

S8. Arrhenius parameters

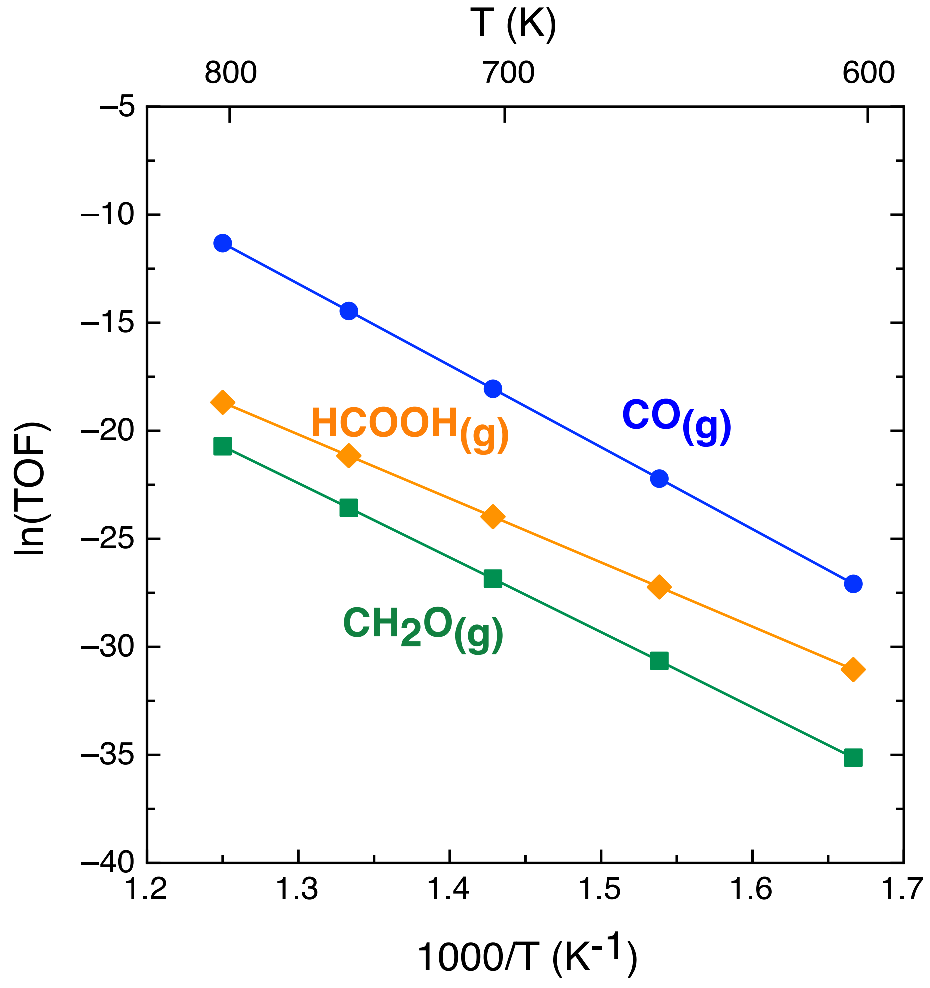

Figure S5. Arrhenius fits of $\ln r_i$ *vs.* $1000 \cdot T^{-1}$ for $CO_{(g)}$, $HCOOH_{(g)}$ and $CH_2O_{(g)}$ production in the range of temperatures 600 K – 800 K.

Table S4. Fitted parameters and associated errors to $\ln r_i = \ln A_i - \frac{E_a}{1000 \cdot R} \cdot \frac{1000}{T}$ for $CO_{(g)}$, $HCOOH_{(g)}$ and $CH_2O_{(g)}$ production in the range of temperatures 600 – 800 K. A_i units are s^{-1}.

Species	$\ln A_i$	$\delta[\ln A_i]$	$\frac{-E_a}{1000 \cdot R}$ / K	$\delta\left[\frac{-E_a}{1000 \cdot R}\right]$ / K	r^2
$CO_{(g)}$	36.002	0.045	-37.844	0.031	1
$HCOOH_{(g)}$	18.418	0.034	-29.677	0.024	1
$CH_2O_{(g)}$	22.569	0.115	-34.608	0.079	1

References

(1) Steinfeld, J.I.; Francisco, J.S.; Hase, W.L. Chemical Kinetics and Dynamics, Prentice Hall, Englewood Cliffs, USA, **1989**.
